# Airway surface hyperviscosity and defective mucociliary transport by IL-17/TNF-**α** are corrected by **β**-adrenergic stimulus

**DOI:** 10.1172/jci.insight.164944

**Published:** 2022-11-22

**Authors:** Daniela Guidone, Martina Buccirossi, Paolo Scudieri, Michele Genovese, Sergio Sarnataro, Rossella De Cegli, Federico Cresta, Vito Terlizzi, Gabrielle Planelles, Gilles Crambert, Isabelle Sermet, Luis J.V. Galietta

**Affiliations:** 1 Telethon Institute of Genetics and Medicine (TIGEM), Pozzuoli, Italy.; 2 U.O.C. Genetica Medica, IRCCS Istituto Giannina Gaslini, Genova, Italy.; 3 Department of Neurosciences, Rehabilitation, Ophthalmology, Genetics, Maternal and Child Health (DINOGMI), University of Genova, Genova, Italy.; 4 Centro Fibrosi Cistica, IRCCS Istituto Giannina Gaslini, Genova, Italy.; 5 Meyer Children’s Hospital, Cystic Fibrosis Regional Reference Center, Department of Paediatric Medicine, Firenze, Italy.; 6 Centre de Recherche des Cordeliers, INSERM UMRS 1138, Sorbonne Université, Université Paris Cité, Paris, France.; 7 CNRS EMR 8228, Paris, France.; 8 Institut Necker Enfants Malades/INSERM U1151, Paris, France.; 9 Department of Translational Medical Sciences (DISMET), University of Napoli “Federico II”, Napoli, Italy.

**Keywords:** Pulmonology, Innate immunity, Ion channels

## Abstract

The fluid covering the surface of airway epithelia represents a first barrier against pathogens. The chemical and physical properties of the airway surface fluid are controlled by the activity of ion channels and transporters. In cystic fibrosis (CF), loss of CFTR chloride channel function causes airway surface dehydration, bacterial infection, and inflammation. We investigated the effects of IL-17A plus TNF-α, 2 cytokines with relevant roles in CF and other chronic lung diseases. Transcriptome analysis revealed a profound change with upregulation of several genes involved in ion transport, antibacterial defense, and neutrophil recruitment. At the functional level, bronchial epithelia treated in vitro with the cytokine combination showed upregulation of ENaC channel, ATP12A proton pump, ADRB2 β-adrenergic receptor, and SLC26A4 anion exchanger. The overall result of IL-17A/TNF-α treatment was hyperviscosity of the airway surface, as demonstrated by fluorescence recovery after photobleaching (FRAP) experiments. Importantly, stimulation with a β-adrenergic agonist switched airway surface to a low-viscosity state in non-CF but not in CF epithelia. Our study suggests that CF lung disease is sustained by a vicious cycle in which epithelia cannot exit from the hyperviscous state, thus perpetuating the proinflammatory airway surface condition.

## Graphical abstract

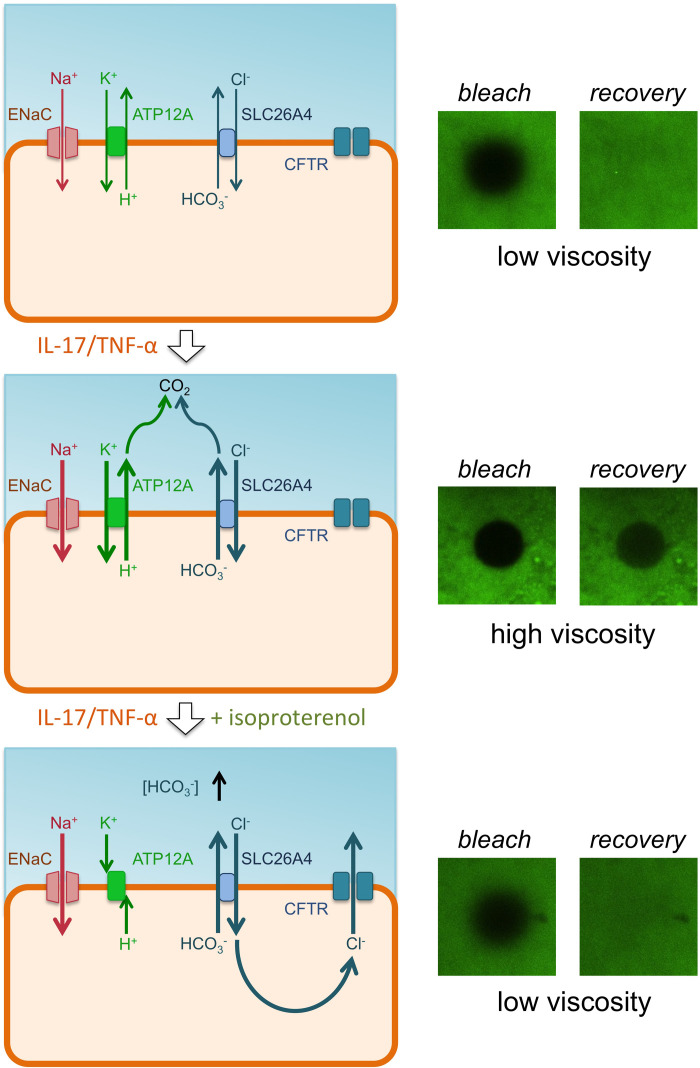

## Introduction

The airway epithelium represents an important barrier toward pathogenic agents that enter the respiratory system with inhaled air (1). One of the intrinsic defense mechanisms adopted by the epithelium is mucociliary clearance (MCC), in which mucus acts as a trap for bacteria, fungi, and viruses, and the beating cilia propel mucus toward oro-pharynx (2, 3). MCC also requires the presence of an airway surface liquid (ASL), whose low viscosity facilitates ciliary beating. The chemical and physical properties of ASL are controlled by the coordinated activity of ion channels and transporters that mediate the transepithelial absorption and secretion of Na^+^, Cl^–^, HCO_3_^–^, and H^+^, as well as those of other solutes (4). ASL also contains antimicrobial molecules such as defensins, lactoferrin, and lysozyme (5). The innate defense mechanisms in the airway epithelium are further modulated during infection and inflammation. ILs, IFNs, and lipids released by cells of the immune system can reshape the transcriptome and proteome of airway epithelial cells to potentiate their defense barrier function, although excessive response may lead to lung damage (6–8). IL-4 and IL-13, which are characteristic of the Th2 immune response, boost mucus production, particularly that of MUC5AC mucin (9, 10), and enhance expression and function of proteins (SLC26A4, ANO1, CFTR, SLC12A2) that mediate anion transport (11, 12). The enhanced anion secretion, particularly that of HCO_3_^–^, could be instrumental in supporting mucus release and expansion (13–15), as well as in supporting innate antimicrobial activity in the ASL (16).

The defense function of the airway epithelium can be compromised in genetic diseases such as cystic fibrosis (CF) and primary ciliary dyskinesia (17, 18). In CF, loss of function of the CFTR channel impairs Cl^–^ secretion. The consequence is the dehydration of the ASL, which results in defective MCC (19, 20). CFTR also contributes to HCO_3_^–^ transport, either directly and/or by secreting Cl^–^ that is then exchanged with HCO_3_^–^ by SLC26A4 (21, 22). The impairment of HCO_3_^–^ secretion may disrupt the activity of antimicrobial molecules and alter the properties and the release of mucus (13–16). The combination of defects caused by lack of CFTR function leads to chronic bacterial infections that, in turn, sustain a severe inflammatory process with mucus hypersecretion. The perturbation of MCC in CF may also be due to enhanced function of ENaC, the epithelial channel that is responsible for Na^+^ and fluid absorption — a process that counteracts secretion by CFTR (23).

It is still not clear to what extent the functional abnormalities occurring in the lungs of CF patients are a direct consequence of CFTR loss of function or the result of subsequent infection and inflammation. In a previous study, we found that ATP12A, a proton pump expressed in the apical membrane (AM) of airway epithelial cells, is upregulated in the lungs of CF patients (24). The activity of ATP12A is considered to be pathogenic, since acidification of the airway surface may abolish antibacterial activity and make mucus secretions more viscous (13–16, 25). We also found that differential ATP12A expression observed in ex vivo samples disappears when CF and non-CF epithelia are examined under sterile culture conditions. ATP12A expression was markedly induced in both cell types following treatment with IL-4 (24). These findings suggest that ATP12A upregulation in vivo is a consequence of inflammation and not a direct effect of CFTR loss. The aim of the present study was to investigate the link between ATP12A and inflammation. By testing different proinflammatory stimuli, we discovered that the treatment of bronchial epithelia in vitro with IL-17A (IL-17) and TNF-α triggers a complex program that, besides upregulating ATP12A, alters the expression and function of multiple channels and transporters with alteration of the chemical and physical properties of airway surface.

## Results

**Figure 1:**
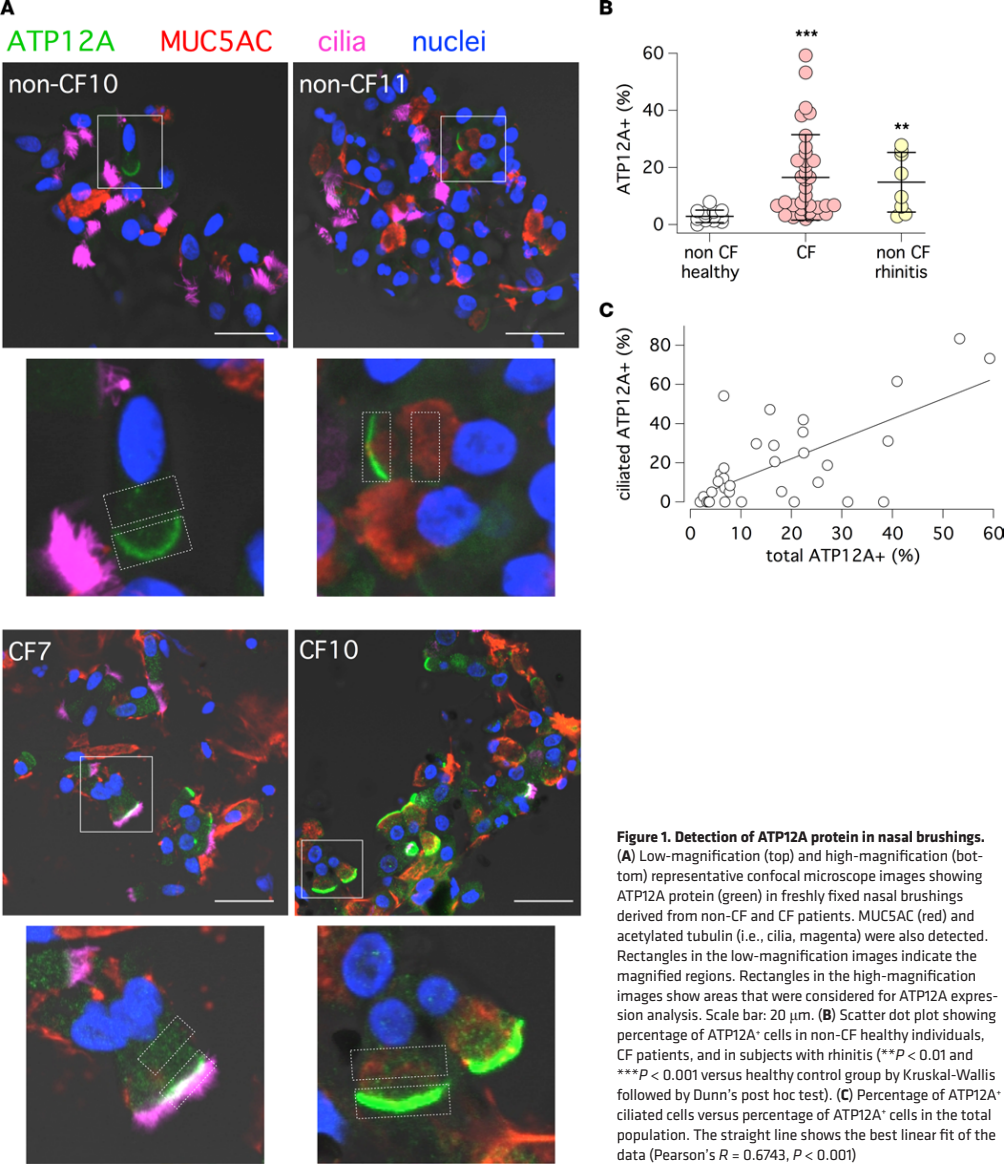
Detection of ATP12A protein in nasal brushings. (**A**) Low-magnification (top) and high-magnification (bottom) representative confocal microscope images showing ATP12A protein (green) in freshly fixed nasal brushings derived from non-CF and CF patients. MUC5AC (red) and acetylated tubulin (i.e., cilia, magenta) were also detected. Rectangles in the low-magnification images indicate the magnified regions. Rectangles in the high-magnification images show areas that were considered for ATP12A expression analysis. Scale bar: 20 μm. (**B**) Scatter dot plot showing percentage of ATP12A^+^ cells in non-CF healthy individuals, CF patients, and in subjects with rhinitis (**P < 0.01 and ***P < 0.001 versus healthy control group by Kruskal-Wallis followed by Dunn’s post hoc test). (**C**) Percentage of ATP12A^+^ ciliated cells versus percentage of ATP12A^+^ cells in the total population. The straight line shows the best linear fit of the data (Pearson’s *R* = 0.6743, *P* < 0.001)

In a previous study, we found strong ATP12A expression in the bronchial epithelium of CF patients undergoing lung transplants (24). We asked whether ATP12A upregulation was a consequence of the advanced stage of the disease. Therefore, we investigated ATP12A expression in the nasal mucosa of CF patients of various age and clinical conditions. For this purpose, we adopted a simple nasal brushing procedure that we recently developed (26). After brushing, detached cells were immediately fixed and processed for immunofluorescence. Figure 1A shows that cells from many CF patients have enhanced expression of ATP12A in the AM. Furthermore, many CF samples showed unusual ATP12A expression in ciliated cells (Figure 1A, CF7 sample). By combining results from 14 non-CF control individuals and 35 CF patients, we found that ATP12A was significantly more expressed in CF, with some patients showing a percentage of ATP12A-expressing cells as high as 50% to 60% (Figure 1B). Interestingly, ATP12A was also significantly upregulated in non-CF individuals with rhinitis at the time of brushing (Figure 1B). Because of the broad range of ATP12A expression in CF samples, we plotted the percentage of ATP12A-expressing cells versus age or pulmonary function indicated by forced expiratory volume in the first second (FEV_1_) (Supplemental Figure 1; supplemental material available online with this article; https://doi.org/10.1172/jci.insight.164944DS1). We found no correlation. There was high ATP12A expression irrespective of age, so that even very young patients (<10 years) had high expression.

ATP12A is typically expressed in nonciliated mucus-producing cells (24). However, it has been shown, by single-cell RNA-Seq (scRNA-Seq), that ATP12A expression can appear in ciliated cells as an early sign of inflammation and transdifferentiation to goblet cells (27). Interestingly, in nasal samples, we found a correlation between ATP12A expression in the global cell population and extent of localization in ciliated cells (Figure 1C). This finding suggests that ATP12A expression is related to the status of mucosal inflammation.

**Figure 2:**
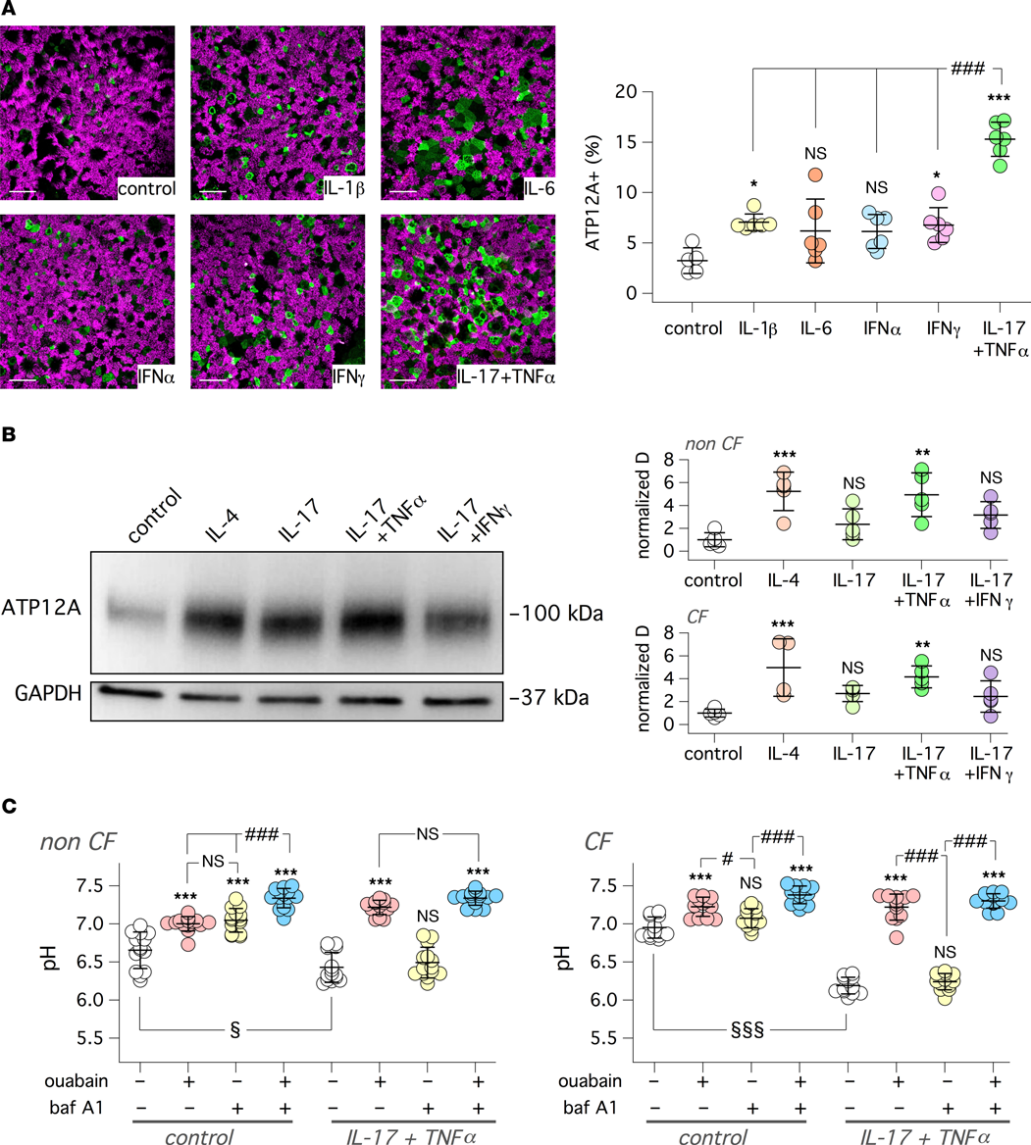
Upregulation of ATP12A expression and function by IL-17/TNF-α in vitro. (**A**) Representative confocal microscope images showing detection of ATP12A (green) and acetylated tubulin (i.e., cilia, magenta). Images are *xy* scans of CF cultured bronchial epithelial cells treated for 72 hours with/without cytokines (10 ng/mL for IL-6, IL-1β, TNF-α, IFN-α, and IFN-γ; 20 ng/mL for IL-17). Scale bar: 25 μm. Scatter dot plot reporting percentage of ATP12A^+^ cells in bronchial epithelia with indicated treatments (*P < 0.05 and ***P < 0.001 versus control; ^###^*P* < 0.001 between indicated conditions; ANOVA and Tukey’s post hoc test). (**B**) Representative image (left) and summary of data (right) deriving from Western blot analysis of ATP12A protein in lysates of bronchial epithelial cells treated with/without single cytokines or cytokine combinations. The scatter dot plots report the band intensity for ATP12A normalized for GAPDH expression. Data were obtained from 5 CF and 5 non-CF bronchial cell preparations (**P < 0.01 and ***P < 0.001 versus control; ANOVA and Dunnett’s post hoc test). (**C**) Apical fluid pH measurement with SNARF-1 dextran probe in non-CF and CF bronchial epithelia. Cells were treated with/without IL-17 + TNF-α. Experiments were done in bicarbonate-free conditions. Where indicated, the apical solution contained ouabain (200 μM), bafilomycin A1 (100 nM), or both compounds together. pH was measured after 3 hours of incubation under CO_2_-free conditions (***P < 0.001 versus control; ^#^*P* < 0.05 and ^###^*P* < 0.001 between indicated conditions; ^§^*P* < 0.05 and ^§§§^*P* < 0.001 for treated versus untreated epithelia; ANOVA and Tukey’s post hoc test).

We treated bronchial epithelia in vitro with a panel of proinflammatory cytokines to further investigate the link between ATP12A expression and inflammation. In particular, we chose IL-1β and IL-17 plus TNF-α, stimuli that are associated with mucus hypersecretion and neutrophilic infiltration, respectively (28, 29). In parallel, we also tested IFN-α, IFN-γ, and IL-6 as representative of other types of proinflammatory stimuli. After treatment (72 hours), ATP12A expression was investigated by immunofluorescence. We found that the IL-17/TNF-α combination was particularly effective in increasing the percentage of cells expressing ATP12A with respect to control and all other treatments (Figure 2A). This effect was also investigated by immunoblot. The IL-17/TNF-α combination, as well as IL-4, significantly increased the ATP12A band in both CF and non-CF epithelia (Figure 2B; see complete unedited blots in the supplemental material).

We evaluated ATP12A function by measuring pH under bicarbonate-free conditions (large volume in situ pH assay). We added the pH-sensitive fluorescent probe SNARF-1 conjugated to high–molecular weight dextran, dissolved in a modified PBS (60 μL, pH 7.3) with low buffer capacity, to the apical side of epithelia. This solution also contained ouabain (ATP12A inhibitor), bafilomycin A1 (V-ATPase inhibitor), both compounds together, or vehicle. The basolateral culture medium was buffered with Hepes (pH 7.4). Fluorescence was recorded and pH was calculated at the starting point and after 1 and 3 hours following probe addition. Figure 2C shows that non-CF and CF epithelia acidify the apical solution after 3 hours. Unexpectedly, acidification was more marked in non-CF epithelia, with pH values close to 6.5 (Figure 2C, left). Inclusion of proton pump inhibitors also led to some unexpected results. In non-CF cells, bafilomycin A1 and ouabain were both effective in partially reducing the acidification, and combination of the 2 compounds had an additive effect. In contrast, CF cells were mostly sensitive to ouabain and not to bafilomycin A1 (Figure 2C, right). Treatment of CF and non-CF epithelia with IL-17/TNF-α induced marked acidification that was exclusively sensitive to ouabain. In fact, the bafilomycin-sensitive component observed in non-CF epithelia under untreated conditions disappeared after cytokine treatment.

**Figure 3:**
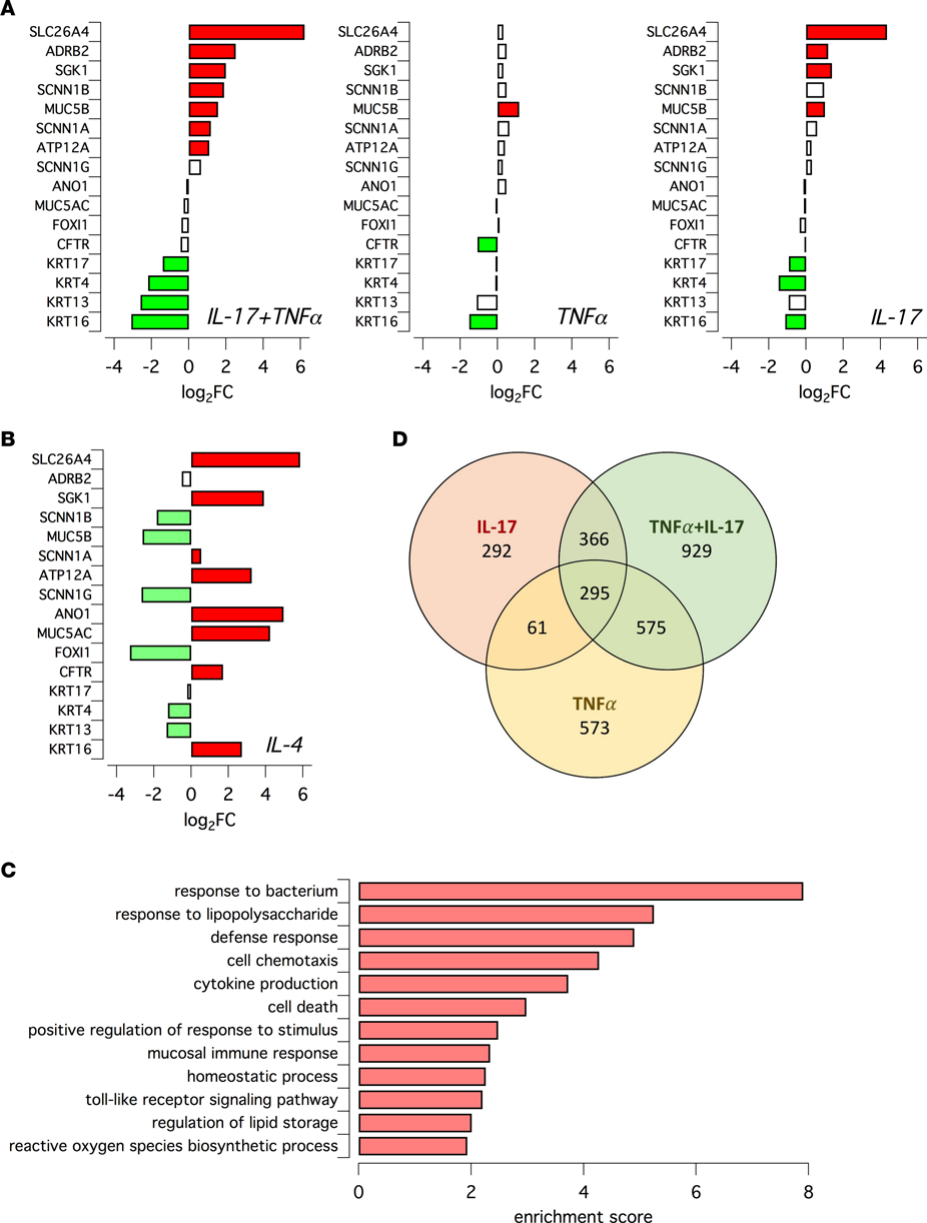
Analysis of gene expression changes in epithelia treated with inflammatory stimuli. (**A** and **B**) Bar graphs reporting the expression changes for indicated genes in epithelia treated with TNF-α + IL-17, TNF-α, IL-17, and IL-4. Data were obtained by RNA-Seq. Red and green indicate statistically significant upregulation and downregulation, respectively. (**C**) Biological processes which were associated with gene expression changes induced by TNF-α/IL-17 treatment. (**D**) Venn diagram showing extent of overlap for genes whose expression was upregulated by indicated cytokine treatment.

We analyzed the effect of IL-17 plus TNF-α at the transcriptome level using the QuantSeq 3′ mRNA-Seq. For comparison, we also investigated the individual effects of IL-17, TNF-α, and IL-4. This analysis revealed a profound change elicited by the IL-17/TNF-α combination, with upregulation of many genes. Supplemental Figure 2 shows data for the 80 top upregulated genes, with SLC26A4 at the top of the list. The extent of upregulation ranged from approximately 60-fold (SLC26A4) to 4-fold (KCNE3). The list includes genes involved in transepithelial transport (SLC26A4, SLC5A8, SLC5A1, SLC6A20), cytokine/chemokine signaling (CSF3, IL-19, CCL20, CXCL6, CXCL5, CXCL1), and modulation of immune response and antimicrobial activity (IDO1, NOS2, DEFB4A, LTF, DEFB4B). A selection of genes, with relevance to the topic of our study — i.e., ion transport and defense function — is shown in Figure 3, A and B. In addition to SLC26A4, the IL-17/TNF-α combination also increased the expression of ADRB2 (the β2-adrenergic receptor), the kinase SGK1, the ENaC subunits SCNN1B and SCNN1A, mucin MUC5B, and ATP12A. The extent of gene expression increase was relatively modest (between 5- and 2-fold) compared with that of the most upregulated genes (Supplemental Figure 2). The effects of IL-17/TNF-α combination for these selected genes were more like those of IL-17 alone (Figure 3A). For example, SLC26A4 was upregulated by IL-17 but not by TNF-α. It is important to note that the analysis of IL-4 treatment revealed important differences (Figure 3B). For example, IL-4 increased the expression of MUC5AC and not of MUC5B, which was instead downregulated (Figure 3, A and B). Also, in agreement with our previous studies (11, 12, 30), IL-4 enhanced the expression of the ANO1 (TMEM16A) Ca^2+^-activated Cl^–^ channel, which was instead unaffected by IL-17/TNF-α. Furthermore, the upregulation of ATP12A was more marked in IL-4–treated epithelia. The transcriptomic changes elicited by IL-17/TNF-α were investigated with functional annotation tools to identify the specific biological processes involved. Enriched processes include the response to bacteria and to bacterial components, cytokine production, mucosal immune response, and TLR signaling pathway (Figure 3C). The Venn diagram in Figure 3D shows the comparison of the 3 gene expression profiles of cells treated with the IL-17/TNF-α in combination and with the single cytokines. The heatmaps in Supplemental Figure 3 (data available in Supplemental Table 1) show that the gene expression changes in CF and non-CF epithelia elicited by IL-17/TNF-α were quite similar.

**Figure 4:**
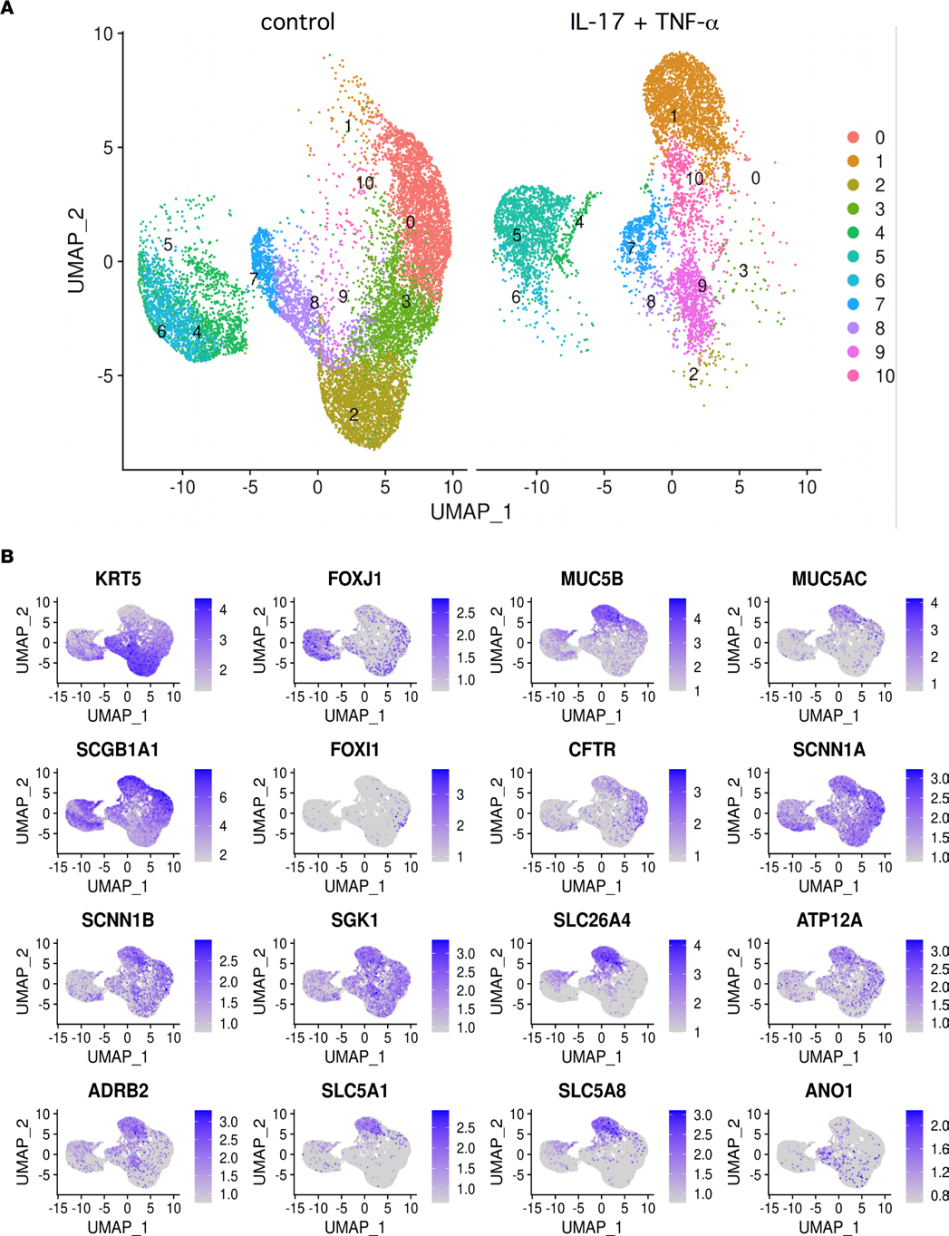
Analysis of epithelial transcriptome by single-cell RNA-Seq. (**A**) Global representation of gene expression in epithelia kept under control conditions (left) or treated with IL-17/TNF-α (right). Each dot represents a single cell, whose position in the 2D map reports the transcriptional similarity with respect to the neighbor cells. The different colors and numbers correspond to cells clusters with similar transcriptome. (**B**) Two-dimensional maps showing the expression of indicated genes within the epithelial cell population. MUC5B, SLC26A4, ATP12A, ADRB2, SLC5A1, and SLC5A8 expression is particularly concentrated in the region corresponding to cluster 1 (**A**), which is enriched by IL-17/TNF-α treatment.

We applied scRNA-Seq to further analyze the effects of IL-17/TNF-α treatment. The images in Figure 4A summarize the gene expression profile under control conditions (left) and after treatment (right). Each dot represents a single cell. Cells with similar transcriptomes are positioned close to each other, forming clusters numbered from 0 to 10. Figure 4B reports the position of cells in the 2D uniform manifold approximation and projection (UMAP) space with expression of particular genes selected because they are either markers of specific cell types (e.g., KRT5 for basal cells, FOXJ1 for ciliated cells, FOXI1 for ionocytes) or because they are modulated by IL-17/TNF-α. The cytokine combination markedly changes gene expression in the epithelium, as highlighted by the nature of the clusters. For example, cluster 2, which corresponds to basal cells (high KRT5 expression), almost does not contain treated cells. Instead, cluster 1 is strongly enriched in treated cells and shows predominant expression of genes induced by IL-17/TNF-α: SLC26A4, MUC5B, ADRB2, and ATP12A (Figure 4B). Also, clusters 4–6 — which, based on FOXJ1 expression, probably represent ciliated cells — show a change in their transcriptional state. Clusters 4 and 6 contain more cells in untreated epithelia, while cluster 5 is enriched in treated epithelia.

**Figure 5:**
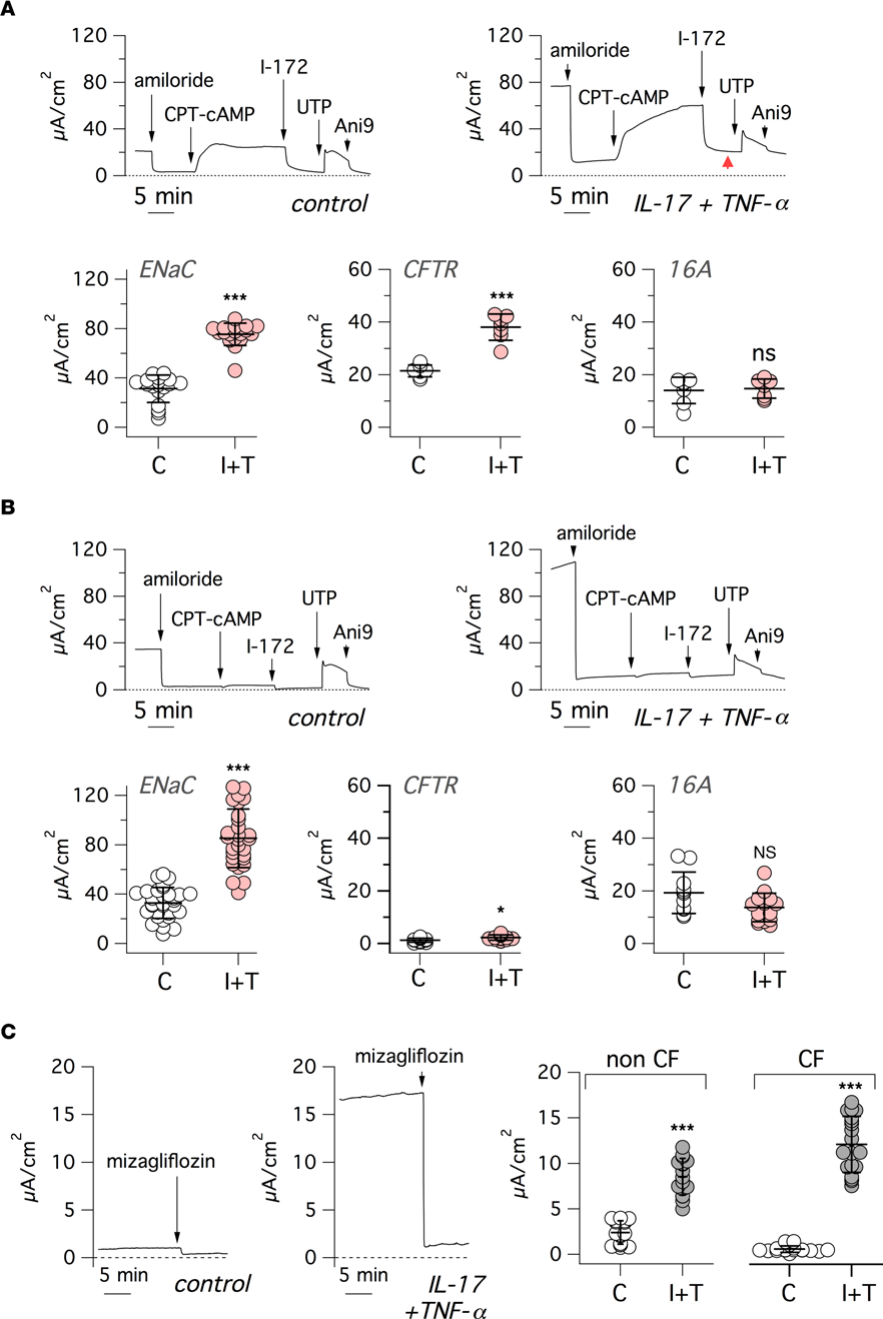
Modification of electrogenic ion transport by IL-17/TNF-α. (**A** and **B**) Representative traces (top) and summary of data (bottom) from short-circuit current recordings done on non-CF (**A**) and CF (**B**) bronchial epithelia, treated with/without IL-17/TNF-α combination. During recordings, epithelia were sequentially exposed to: amiloride (10 μM), CPT-cAMP (100 μM), CFTR_inh_-172 (I-172, 20 μM), UTP (100 μM), and Ani9 (5 μM). Red arrowhead: higher residual current in cytokine treated epithelia. The scatter dot plots report, for control and IL-17/TNF-α–treated(I+T–treated) epithelia, the amplitude of amiloride, CFTR_inh_-172, and UTP effects, which are representative of ENaC, CFTR, and TMEM16A function, respectively (*P < 0.05; **P < 0.01; and ***P < 0.001 versus control; Mann-Whitney *U* test). (**C**) Representative short-circuit current recordings (left) and summary of data (right) showing effect of mizagliflozin (10 μM) on control and I+T–treated epithelia. Mizagliflozin was added in the presence of amiloride, CFTR_inh_-172, and Ani9. (***P < 0.001 versus control; Mann-Whitney *U* test).

We carried out short-circuit current recordings on non-CF and CF epithelia to evaluate the effect of IL-17/TNF-α on electrogenic ion transport (Figure 5, A and B). During recordings, we sequentially added: (a) amiloride (10 μM), to block ENaC-dependent Na^+^ absorption; (b) CPT-cAMP (100 μM) followed by CFTR_inh_-172 (10 μM) to activate and inhibit CFTR, respectively; and (c) UTP (100 μM) and Ani9 (10 μM) to induce and block Ca^2+^-activated Cl^–^ secretion through TMEM16A (ANO1). Treatment with IL-17/TNF-α induced a significant increase in ENaC activity (i.e., the amplitude of amiloride effect) in both CF and non-CF epithelia. This effect was unexpected, since other cytokines — namely, IL-4, IL-13, and IL-1β — actually decrease ENaC activity (31–33). CFTR current was also increased by IL-17/TNF-α, as recently reported (34), and this effect could be even detected in CF epithelia, despite the very low level of CFTR function. Instead, the response to UTP was not increased, in agreement with unaltered TMEM16A gene expression.

It can be noticed in the representative traces shown in Figure 5, A and B, that epithelia treated with IL-17/TNF-α show higher levels of basal current that persist even after addition of amiloride and CFTR_inh_-172 (Figure 5A, red arrowhead). This current was also resistant to Ani9, an effective blocker of TMEM16A (Figure 5, A and B). We hypothesized that the baseline current elicited by the cytokine combination is due to another electrogenic transport, possibly Na^+^-dependent glucose uptake at the AM. Indeed, the mRNA for SLC5A1 (also referred to as SGLT1, the sodium/glucose cotransporter) was upregulated (8-fold; Supplemental Figure 2) by IL-17/TNF-α. Accordingly, we tested mizagliflozin, a potent and selective inhibitor of SLC5A1. This compound caused a large drop of the baseline current in cytokine treated epithelia (Figure 5C). In fact, the currents remaining after mizagliflozin addition in control and cytokine-treated epithelia were comparable.

**Figure 6:**
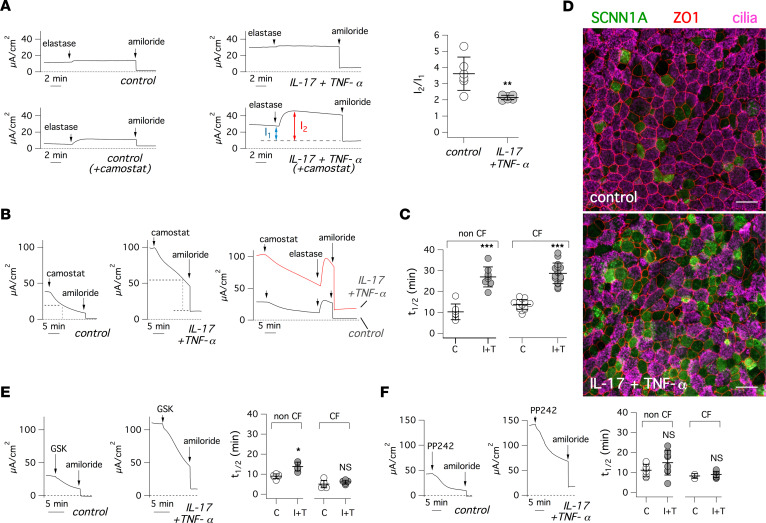
Mechanism of ENaC upregulation by IL-17/TNF-α. (**A**) Representative short-circuit current traces (left) and summary of data (right) for experiments on epithelia treated with/without IL-17/TNF-α for 72 hours. During recordings, elastase (1.5 μM) and amiloride (10 μM) were sequentially added. Where indicated, epithelia were apically treated with camostat (3 μM) for 18 hours. The scatter dot plot shows, for experiments with camostat pretreatment, the ratio I2/I1, where I1 and I2 are the current amplitudes before and after elastase, respectively (**P < 0.01; Student’s *t* test). (**B**) Representative traces from experiments with/without IL-17/TNF-α treatment in which camostat (3 μM) and amiloride were sequentially added. Where indicated, experiments also included addition of elastase (1.5 μM) after camostat. (**C**) Summary of data showing the rate of ENaC current decay after camostat addition (C, control; I+T, IL-17/TNF-α). Data (t_1/2_) report the time at which the current decayed to half of initial amplitude (***P < 0.001; Student’s *t* test). (**D**) Representative confocal microscope images, from control- and IL-17/TNF-α–treated epithelia, in which SCNN1A, ZO-1, and cilia were detected by immunofluorescence. Scale bar: 25 μm. (**E** and **F**) Representative short-circuit current traces and summary of data for experiments where control- and IL-17/TNF-α–treated epithelia were apically exposed to 25 μM GSK650394 (**E**) or 5 μM PP242 (**F**) before amiloride. The scatter dot plots report the t_1/2_ values for each condition. (*P < 0.05 versus control; Student’s *t* test).

We were intrigued by the marked upregulation of ENaC function elicited by IL-17/TNF-α. Since the increase in SCNN1A and SCNN1B transcripts was small (Figure 3A), we considered the possibility of a posttranscriptional mechanism. ENaC activity is known to be regulated by proteolytic activation (35, 36). Therefore, we tested the effect of elastase (Figure 6A, top traces). Elastase was ineffective on epithelia kept under control conditions or treated with IL-17/TNF-α, indicating that ENaC is fully cleaved in both conditions and that the effect of the cytokine combination is not mediated by an increase in ENaC proteolysis. In parallel experiments, we incubated epithelia for 18 hours with the protease inhibitor camostat, which is effective on the proteases that regulate ENaC (37). As expected, camostat treatment reduced the size of ENaC currents and subsequent addition of elastase to camostat-treated epithelia resulted in rapid activation of currents (Figure 6A, bottom traces). The activating effect of elastase demonstrates that camostat reduces ENaC activity by preventing its proteolysis. Interestingly, we noticed a difference in the fraction of the total current that was sensitive to elastase treatment. This parameter was smaller in epithelia treated with IL-17/TNF-α (Figure 6A).

Since the ENaC turnover at the plasma membrane — dependent on channel insertion, proteolytic activation, and internalization — is quite fast (38, 39), we also added camostat during recordings of epithelia previously treated with/without IL-17/TNF-α (Figure 6B). Camostat caused a rapid reduction in transepithelial current. We added amiloride during this decay phase. There was a sharp drop of the current that then reached a stable level. This behavior indicates that the current rundown caused by camostat is based on ENaC inhibition; otherwise, it would have continued in the presence of amiloride. Importantly, we noticed a difference in the time course of the decay between control- and IL-17/TNF-α–treated cells. The time required to reach half of the initial amplitude in control-treated epithelia was 10–14 minutes, a value comparable with what was previously reported: 11–17 minutes (38, 39). With IL-17/TNF-α, the decay was instead significantly slower, with a mean half-time of ~28 minutes in both CF and non-CF epithelia (Figure 6C). It is also interesting to note that the trend of the camostat-induced decay is different between control- and IL-17/TNF-α–treated epithelia. In the control condition, the decay appears to follow a single exponential fashion. With the cytokine combination, the decay appears biphasic, with an initial small and relatively rapid decay followed by a large and slower linear phase. The representative immunofluorescence images in Figure 6D show that SCNN1A protein is indeed increased on the apical side of cells treated with IL-17/TNF-α.

Internalization and degradation of ENaC is blocked by SGK1-dependent phosphorylation (40), which prevents ENaC ubiquitination by NEDD4L. Since SGK1 was one of the genes upregulated by IL-17/TNF-α (Figure 3A), we used a SGK1 inhibitor to assess the effect on ENaC activity (Figure 6E). GSK650394 caused a rapid rundown of ENaC currents. The rundown was not significantly different between control- and IL-17/TNF-α–treated CF epithelia and only modestly different in non-CF epithelia. Furthermore, elastase was ineffective when added during the GSK650394-induced current rundown (Supplemental Figure 4), indicating that the inhibitor is acting downstream from the proteolytic step. We also inhibited mTORC2, since this regulatory complex acts upstream from SGK1 to control ENaC function (41, 42). The mTORC2 inhibitor PP242 also caused ENaC rundown, and the kinetics between control- and cytokine-treated epithelia were not significantly different (Figure 6F). These results support SGK1 upregulation as the mechanism responsible for the enhanced ENaC function in epithelia exposed to IL-17/ TNF-α. Indeed, SGK1 inhibition cancels the difference between control and treated epithelia.

**Figure 7:**
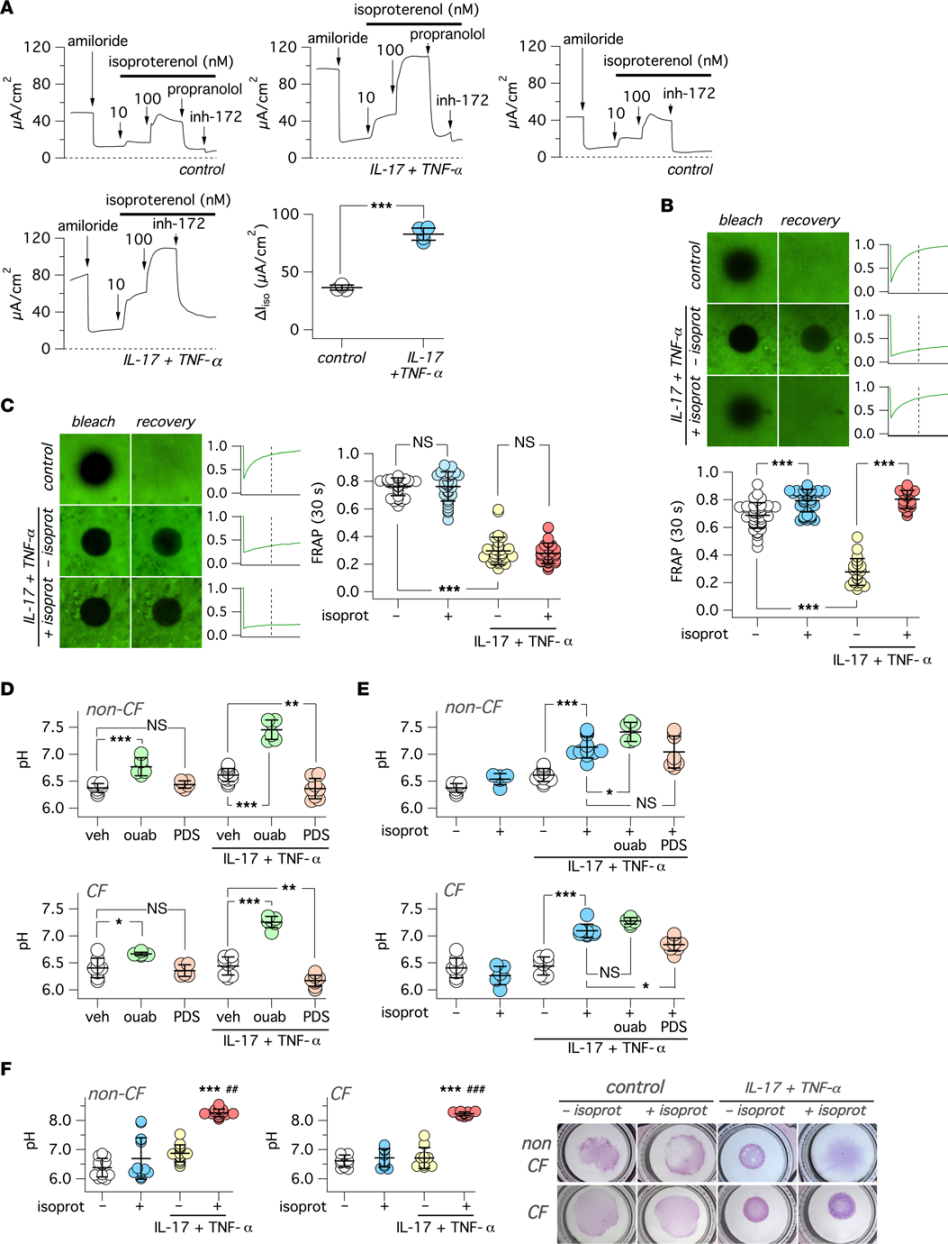
Effect of β-adrenergic stimulus on airway surface properties. (**A**) Representative traces and summary of data for short-circuit current experiments on epithelia treated with/without IL-17/TNF-α, in which isoproterenol (10 and 100 nM) was added to stimulate CFTR activity. Where indicated CFTR_inh_-172 (20 μM) and propranolol (5 μM) were also added. The scatter dot plot shows the amplitude of CFTR_inh_-172 effect in epithelia previously stimulated with isoproterenol. ***P < 0.001 versus control (Student’s *t* test). (**B** and **C**) Data from FRAP experiments on non-CF (**B**) or CF (**C**) epithelia treated with/without IL-17/TNF-α (72 hours). Where indicated, isoproterenol (100 nM) was added for 3 hours on the basolateral side before experiments. Representative images show the epithelial surface, labeled with FITC-dextran, after photobleaching of a circular area and then after a 60-second recovery time. The traces on the side of each image pair report the time course of fluorescence recovery. The vertical dashed line indicates the 30-second time point. Scatter dot plots show the normalized fluorescence value measured at 30 seconds after photobleaching. ***P < 0.001 (Kruskal-Wallis and Dunn’s test). (**D** and **E**) Measurement of pH in the apical solution (large volume ex situ pH assay) for non-CF (top) and CF (bottom) epithelia, treated with/without IL-17/TNF-α. Where indicated, the apical solution contained ouabain (ouab, 200 μM) or PDS_inh_-A01 (PDS, 25 μM), whereas the basolateral solution included isoproterenol (100 nM). *P < 0.5; **P < 0.01; and ***P < 0.001 (ANOVA with Tukey’s post hoc test). (**F**) Measurement of pH on the apical surface (small volume in situ pH assay) in non-CF and CF epithelia treated with/without IL-17/TNF-α (72 hours). Where indicated, the basolateral solution included isoproterenol (100 nM, for 3 hours before experiment). The scatter dot plots show pH values for the indicated conditions. ***P < 0.001 versus untreated cells (no cytokine treatment, no isoproterenol). ^##^*P* < 0.01; ^###^*P* < 0.001 versus cells with cytokine treatment/without isoproterenol (Kruskal-Wallis and Dunn’s test). The images show the pattern of SNARF-1 dextran distribution on epithelial surface for the indicated conditions.

We were also interested in investigating the consequences of ADRB2 transcript upregulation by IL-17/TNF-α (Figure 3A). ADRB2 activation results in intracellular cAMP elevation, which should, in turn, activate CFTR. We carried out short-circuit current recordings, during which the ADRB2 agonist isoproterenol was added on the basolateral side of epithelia. Addition of 10 and 100 nM of isoproterenol elicited rapid activation of currents that could be similarly blocked by CFTR_inh_-172 and by the ADRB2 antagonist propranolol (Figure 7A). Epithelia treated with IL-17/TNF-α showed a more than 2-fold increase in isoproterenol-induced currents.

Given the alteration by IL-17/TNF-α of multiple mechanisms involved in controlling airway surface chemical/physical properties and MCC (ENaC-dependent Na^+^ absorption, CFTR-dependent anion secretion, ATP12A-dependent proton secretion), we carried out a series of functional assays to define the overall effect. First, we evaluated the transport of microbeads on the apical surface. Black microbeads dispersed in a small volume of saline solution were deposited on epithelial surface, and MCC was determined by time-lapse microscopy. Under control conditions, microbeads visibly moved with a velocity of approximately 10 μm/second in both CF and non-CF epithelia (Supplemental Figure 5). Instead, epithelia treated for 72 hours with IL-17/TNF-α showed a near arrest of microbead transport. Importantly, mucociliary transport was resumed when non-CF epithelia treated with the cytokine combination were stimulated for 3 hours with basolateral isoproterenol. In contrast, despite the addition of the β-adrenergic agonist, microbeads remained static in CF epithelia (Supplemental Figure 5).

To clarify the reason for the arrest of microbead transport, we investigated the properties of the apical surface with the Fluorescence Recovery After Photobleaching (FRAP) technique. To apply FRAP, we needed a short distance between the objective and the epithelium, which was impeded by the narrow cup-like shape of the porous support (Snapwell) used to generate the epithelia. To overcome this limitation, we seeded epithelial cells on the opposite side of the porous membrane. To obtain the air-liquid interface (ALI) condition, the medium was removed from the bottom part. Control short-circuit recordings showed that, under this upside-down configuration, cells are still able to form tight epithelia with normal ion transport properties. We added FITC-dextran dissolved in a small volume (5 μL) of saline solution and waited for 3 hours. Representative images in Figure 7, B and C, show that photobleaching was followed by a fast recovery in CF and non-CF epithelia kept under untreated conditions. After 72 hours of treatment with IL-17/TNF-α, we observed a dramatic slowing down of fluorescence recovery. In most cases, there was nearly no recovery at all. Importantly, when we added isoproterenol to the basolateral (top) side, we observed in non-CF epithelia a significant restoration of fluorescence recovery (Figure 7B). In contrast, CF epithelia did not respond to isoproterenol (Figure 7C).

We investigated the effect of IL-17/TNF-α and isoproterenol on apical pH level using conditions that included HCO_3_^–^. In one set of experiments, we added a fixed volume (200 μL) of saline solution to the apical side of epithelia (large volume ex situ pH assay). We also added mineral oil to minimize exchange of CO_2_ between the solution and the atmosphere. After 6 hours, the aqueous apical solution was recovered to measure pH with SNARF-1 dextran conjugate. We found that the solution was essentially acidic, with a pH value close to 6.3 in both CF and non-CF epithelia (Figure 7D). The pH only slightly changed by IL-17/TNF-α treatment. However, in agreement with ATP12A upregulation, epithelia treated with the cytokine combination showed a large alkalinization (approximately 0.9–1.0 pH units) when exposed to ouabain (Figure 7D). Epithelia treated with IL-17/TNF-α also responded to the SLC26A4 inhibitor PDS_inh_-A01 (43), with a small (approximately 0.2–0.3 pH units) but significant acidic shift in pH (Figure 7D). These results indicate that both ATP12A and SLC26A4 are involved in setting the apical pH, possibly with a larger contribution by ATP12A. Surprisingly, stimulation of epithelia with isoproterenol elicited a significant alkalinization (approximately 0.6 pH units) in both CF and non-CF epithelia previously treated with IL-17/TNF-α (Figure 7E). The alkalinization by isoproterenol was significantly reduced by PDS_inh_-A01 in CF epithelia and showed no additivity with ouabain (Figure 7E). We tested YS-01 as a second type of SLC26A4 inhibitor (44). We compared side-by-side PDS_inh_-A01 and YS-01 on SLC26A4 transport with a functional assay in FRT cells (Supplemental Figure 6, A and B). Both compounds were effective, although YS-01 appeared more potent, with near total SLC26A4 inhibition at 5 μM (Supplemental Figure 6, A and B). We tested the effect of YS-01 on the apical pH of epithelia treated with IL-17/TNF-α (Supplemental Figure 6, C and D). YS-01 generated a significant acidic shift in both CF and non-CF epithelia (Supplemental Figure 6, C and D). A similar effect was also seen in CF but not in non-CF epithelia after stimulation with isoproterenol.

We asked whether the alkalinization by isoproterenol is due to SLC26A4 upregulation or ATP12A inhibition. We reasoned that, if isoproterenol increases the activity of SLC26A4, we should have seen a significant change in pH in the presence of ouabain. However, this was not the case, since the pH values with ouabain plus/minus isoproterenol were essentially identical (Supplemental Figure 7, A and B). Furthermore, isoproterenol also caused a significant alkalinization under HCO_3_^–^-free conditions, which excludes SLC26A4 contribution (Supplemental Figure 7, C and D). Such results indicate that the effect of isoproterenol on pH is due to inhibition of ATP12A.

In a second set of experiments, to measure apical pH in a more physiological context, we “stained” the apical surface with a small volume (5 μL) of saline solution containing SNARF-1 dextran (small volume in situ pH assay). With this technique, we could confirm that isoproterenol caused significant alkalinization of the apical surface in CF and non-CF epithelia (Figure 7F). While carrying out these experiments, we noticed a peculiar behavior of SNARF-1 dextran whose distribution, as a colored pattern, could be easily detected by the naked eye and with a low magnification stereomicroscope. In epithelia kept under control conditions, the probe appeared to diffuse on a large area of the epithelium (Figure 7F). In epithelia treated with IL-17/TNF-α, the probe remained confined in a smaller circular area characterized by very sharp borders. In non-CF epithelia, stimulation with isoproterenol reversed this situation, allowing rapid diffusion of the probe over the epithelium (Figure 7F). In CF epithelia, isoproterenol was ineffective.

**Figure 8:**
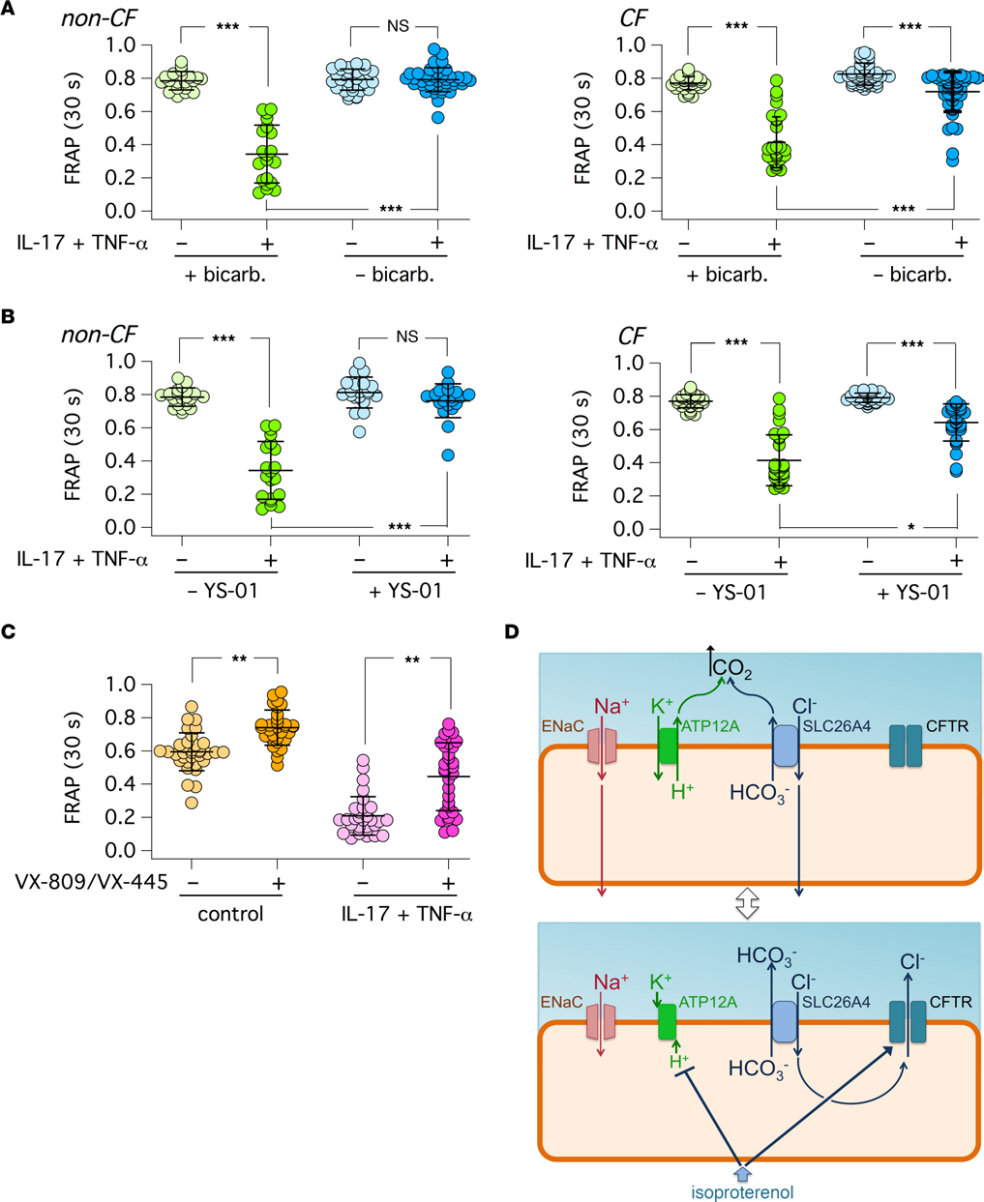
Effect of HCO_3_^–^ transport and CFTR rescue on airway surface properties. The graphs show the results of FRAP experiments on bronchial epithelia that were pretreated for 72 hours with/without IL-17/TNF-α. (**A**) Data obtained in non-CF and CF bronchial epithelia with HCO_3_^–^ or Hepes buffered media on the basolateral side (for 3 hours before measurements). (**B**) Data from experiments in non-CF and CF bronchial epithelia with YS-01 (5 μM) added to the basolateral side for 3 hours before measurements. (**C**) Results from F508del/F508del CF bronchial epithelia. Where indicated, epithelia were treated with the VX-809 (1 μM) plus VX-445 (5 μM) or vehicle in the last 24 hours before measurements. All epithelia were stimulated with isoproterenol (100 nM, basolateral). *P < 0.05; **P < 0.01; and ***P < 0.001 (Kruskal-Wallis and Dunn’s test). (**D**) The illustration shows the proposed changes in ion transport that occur when epithelia treated with IL-17/TNF-α are stimulated with the β-adrenergic agonist.

We asked how HCO_3_^–^ transport affects the properties of airway surface in epithelia treated with IL-17/TNF-α. Therefore, we carried out, in parallel, FRAP experiments in the presence and absence of HCO_3_^–^ in the basolateral medium, with 5% CO_2_ and pure air in the atmosphere, respectively. Surprisingly, the apical surface of epithelia treated with the cytokine combination remained fluid under HCO_3_^–^-free conditions, whereas epithelia in the presence of HCO_3_^–^ developed the expected viscous state (Figure 8A). We also carried out experiments with YS-01 to block SLC26A4 function. YS-01 decreased viscosity in both non-CF and CF epithelia treated with IL-17/TNF-α, indicating that SLC26A4 has an important role in establishing the viscous state by the cytokine combination (Figure 8B). Finally, we asked whether mutant CFTR rescue in CF epithelia with pharmacological correctors of F508del mutation (17) is effective in decreasing apical viscosity. We treated epithelia for 72 hours with/without IL-17/TNF-α. In the last 24 hours, epithelia were treated with the corrector combination VX-809/VX-445 or with vehicle. Then, all epithelia were stimulated with isoproterenol. CF epithelia treated with IL-17/TNF-α and vehicle showed the expected slow fluorescence recovery. However, epithelia that were pretreated with correctors showed a significant improvement in airway surface properties, as indicated by faster fluorescence recovery (Figure 8C).

## Discussion

The initial aim of our study was to elucidate the conditions that lead to ATP12A upregulation in the airways of CF patients (24). By analyzing nasal samples, we confirmed ATP12A upregulation in CF patients, even in individuals of very young age. We found a broad range of values that could be explained with different levels of nasal mucosa inflammation at the time of cell collection. Indeed, there was a positive correlation between ATP12A expression and ATP12A localization in ciliated cells (27).

We tested various types of stimuli in vitro in order to mimic the inflammation occurring in CF airways. We found that IL-17/TNF-α, a stimulus that is associated with bacterial infection and neutrophilic infiltration (29), is particularly effective in enhancing ATP12A expression and function. Our pH measurements in the apical compartment under HCO_3_^–^-free conditions confirmed upregulation of ATP12A at the functional level, as indicated by strong enhancement of the ouabain-sensitive component. It has been shown that airway epithelia, particularly of distal bronchi, display proton secretion mediated by the electrogenic V-ATPase (45). This activity could be dependent on CFTR that, by transporting Cl^–^, provides the required counter ion required for electroneutrality. In agreement with this notion, we found that a bafilomycin-sensitive component was present in non-CF but not in CF epithelia. This finding probably explains why pH was actually more acidic in non-CF epithelia under resting conditions. Interestingly, the bafilomycin-sensitive proton secretion disappeared in non-CF epithelia after treatment with IL-17/TNF-α.

Surprisingly, IL-17/TNF-α treatment also affected other transepithelial transport mechanisms, including ENaC-dependent Na^+^ absorption. ENaC function is regulated at the posttranscriptional level by a complex process involving trafficking to the plasma membrane, channel activation by proteolysis, and fast internalization/degradation by a ubiquitin-dependent mechanism (35, 36, 38, 39, 46, 47). ENaC ubiquitination and internalization is prevented by SGK1-dependent phosphorylation (40). During short-circuit current recordings, we added camostat to inhibit endogenous proteases involved in ENaC activation. Camostat addition caused a rapid rundown of ENaC currents. We interpret this rundown as caused by a lack of compensation for the rapid removal of ENaC from the cell surface by proteolytic activation of new channels delivered to the plasma membrane. Importantly, ENaC function rundown was significantly slower in epithelia treated with IL-17/TNF-α. This finding indicates that ENaC upregulation elicited by the cytokine treatment may depend on a slower rate of internalization. In this respect, one of the genes induced by IL-17/TNF-α is SGK1. In our experiments, an SGK1 inhibitor elicited a rapid rundown of ENaC currents. Furthermore, inhibition of mTORC2, a complex that regulates ENaC through SGK1 (41, 42), also caused rundown of ENaC currents. Such results suggest that SGK1 upregulation may be responsible, at least in part, for the higher ENaC function in epithelia treated with IL-17/TNF-α. To our knowledge, our findings are the first to show upregulation of ENaC by a proinflammatory stimulus. With other cytokines, including IL-4, IL-13, and IL-1β, the effect was actually inhibitory (31, 33). The enhancement of ENaC function by IL-17/TNF-α is expected to worsen the impairment of MCC caused in CF by CFTR loss of function. Enhanced activity of ENaC in CF airways, with the consequent hyperabsorption of Na^+^ and fluid, has been a debated topic (48–52). Our findings reveal a mechanism to explain ENaC upregulation in CF, not directly linked to CFTR defect but to the associated inflammation, and provide a further rationale for the use of ENaC inhibitors to treat CF patients (23).

As previously reported, treatment with IL-17 or IL-17/TNF-α strongly enhances the expression of the SLC26A4 Cl^–^/HCO_3_^–^ exchanger (21, 22, 53). Our work shows that IL-17/TNF-α also affects ATP12A. We carried out pH measurements in the presence of HCO_3_^–^ to assess the role of ATP12A and SLC26A4. In agreement with ATP12A and SLC26A4 upregulation, epithelia treated with IL-17/TNF-α showed alkalinization and acidification with ouabain and SLC26A4 inhibitors, respectively. These results indicate that both transporters contribute to setting apical pH. Interestingly, isoproterenol caused a marked alkalinization, which was of similar amplitude in both CF and non-CF epithelia and which, in part, resembled that produced by ouabain. We reasoned that the increase in pH by isoproterenol could either be caused by SLC26A4 activation or ATP12A inhibition. Our results are in favor of the second type of mechanism, since the effects of isoproterenol and ouabain on pH were not additive. The alkalinization by isoproterenol should then derive from downregulation of ATP12A activity that would uncover SLC26A4-dependent HCO_3_^–^ export. In this respect, we found that the SLC26A4 inhibitor indeed lowered pH in CF epithelia stimulated with isoproterenol. This effect was much lower in non-CF epithelia, possibly suggesting that another mechanism, perhaps CFTR, is contributing to HCO_3_^–^ secretion when SLC26A4 is inhibited.

We asked how the different processes altered by IL-17/TNF-α affect the properties of the airway surface. Measurements of mucociliary transport with microbeads revealed a profound inhibition induced by the cytokine treatment. Importantly, the transport of microbeads was resumed in non-CF epithelia stimulated with a β-adrenergic agonist. Such changes were in agreement with the properties of the apical surface measured with the FRAP technique. Indeed, the diffusion of the high–molecular weight fluorescent dextran was nearly abolished in epithelia treated with IL-17/TNF-α, but it was recovered, only in non-CF epithelia, by isoproterenol stimulation. Such results suggest that impairment of mucociliary transport in epithelia treated with the cytokine combination is caused by hyperviscosity and, possibly, dehydration of airway surface. This condition could result from the combination of enhanced Na^+^ absorption and apical surface acidification due to ENaC and ATP12A upregulation, respectively. In this regard, it should be noted that ATP12A upregulation by IL-13 was previously found to be associated with airway surface hyperviscosity (54). However, in our experiments, isoproterenol elicited alkalinization of apical fluid also in CF epithelia, but it did not affect viscosity. Therefore, the increase in pH is not able per se to make airway surface less viscous. In fact, the recovery of mucociliary transport elicited by isoproterenol only in non-CF epithelia implies that CFTR is endowed with a key function that controls the overall effect of cytokine treatment.

Our experiments that were initially guided to understand the basis of ATP12A upregulation in CF airways have revealed that IL-17/TNF-α is a potent stimulus that, besides affecting ATP12A protein expression and function, modifies other processes with high relevance to airway epithelium barrier function. Importantly, IL-17 is a cytokine whose role in CF lung disease is becoming increasingly apparent. In particular, it has been recently proposed that release of IL-17 from innate lymphoid cells is an important factor responsible for neutrophilic infiltration, inflammation, and lung damage in CF patients (55).

As an attempt to explain the overall output of the complex changes induced by IL-17/TNF-α, we propose the model depicted in Figure 8D, which is based on previous knowledge and results obtained in our study. This model integrates the coordinated activity of various ion channels and transporters (ENaC, ATP12A, SLC26A4) with a key role of CFTR and β-adrenergic stimulus in switching the epithelium between an absorptive and a secretory state. As found in the distal nephron, we propose that ENaC and SLC26A4 work in parallel to generate a net absorption of NaCl (56). Indeed, the absorption of Na^+^ through ENaC is followed by the uptake of Cl^–^ mediated by SLC26A4. In this respect, Haggie and colleagues found that pharmacological inhibition of SLC26A4 results in decreased fluid absorption in airway epithelia (43). In agreement with this study, we found that SLC26A4 inhibition decreased apical viscosity in FRAP experiments. The activity of SLC26A4 implies that HCO_3_^–^ is released on the apical side. Excessive accumulation of HCO_3_^–^, which could actually reverse the direction of transported anions by SLC26A4, should be prevented to support NaCl absorption. Accordingly, the upregulation of ATP12A-dependent proton pumping could be a way to neutralize extruded HCO_3_^–^. The combined secretion of H^+^ and HCO_3_^–^ would push the production of CO_2_ that leaves the apical surface to airway lumen. It is also possible that ATP12A proton pumping, by preventing excessive alkalinization, sustains the activity of ENaC that is favored by acidic pH (57). In summary, the upregulation of ENaC, SLC26A4, and ATP12A by IL-17/TNF-α would be instrumental to promote NaCl/fluid absorption. In this respect, the presence of HCO_3_^–^ appears to be crucial for fluid absorption, since in its absence, we found that the apical surface remains fluid despite the treatment with the cytokine combination. The requirement of HCO_3_^–^ to generate the viscous state is unexpected and prompts reconsideration of the role of this anion in the homeostasis of airway surface properties.

Importantly, we found that the absorptive state induced by IL-17/TNF-α is significantly changed by the β-adrenergic stimulus. Isoproterenol decreased the viscosity of apical surface in non-CF epithelia treated with IL-17/TNF-α. The lack of effect of isoproterenol in CF epithelia highlights the key role of CFTR in this process. Also, we obtained results suggesting that the β-adrenergic stimulus inhibits ATP12A activity. To integrate these results, we postulate that isoproterenol switches the epithelium state from absorptive to secretory. Indeed, the opening of CFTR channel would allow Cl^–^ efflux (Figure 8D). Therefore, Cl^–^ entering the cell by SLC26A4 would be recycled back to the apical surface. The net effect of CFTR and SLC26A4 working in parallel would be the enhanced secretion of HCO_3_^–^. We cannot exclude that part of HCO_3_^–^ secretion is also directly mediated by CFTR. Coupled to ATP12A inhibition, this would result in accumulation of HCO_3_^–^. This is expected to improve the fluidity of the apical surface and to promote antimicrobial activity (13–16, 25). To further support this model, additional evidence will need to be provided in future experiments. In particular, other pharmacological inhibitors of ATP12A, with improved selectivity with respect to ouabain, need to be found. Also, methods to measure local changes in HCO_3_^–^ concentration will need to be developed.

In conclusion, we found that IL-17/TNF-α treatment induces a complex program that involves profound changes in ion transport mechanisms with alteration of airway surface properties. Such alterations may also involve changes in solid content and Ca^2+^ concentration, parameters that did not evaluate in our study. IL-17/TNF-α is associated with bacterial infection and neutrophilic infiltration (29). The increased viscosity of the apical surface by IL-17/TNF-α treatment appears to be detrimental, since mucociliary transport is believed to be beneficial as an antibacterial defense mechanism. However, it can be speculated that transient and local immobilization of bacteria, which would result from increased viscosity, could be useful to avoid dispersion of bacteria over large surfaces and to promote killing by localized epithelium- and neutrophil-dependent mechanisms. In this respect, we found that IL-17/TNF-α enhances the expression of potential antimicrobial genes (SLC5A1, defensins, NOS2, LTF, IDO1). When needed, the absorptive state induced by IL-17/TNF-α would be turned off by the β-adrenergic stimulus to restore mucociliary transport. This switch would be disrupted in CF airways, thus triggering a cycle of inflammation and mucociliary transport impairment. Importantly, our experiments with CFTR correctors show that rescue of F508del-CFTR function allows CF epithelia to acquire, at least in part, the low-viscosity state associated with β-adrenergic stimulus, potentially breaking the damaging cycle. It would be important to find out how the persistent hyperviscous state can be corrected in CF patients with undruggable CFTR mutations. Our results indicate that SLC26A4 inhibition could be an effective approach that could improve MCC in such patients. Our findings are also of high relevance to clarify the pathogenic mechanisms and to find possible therapeutic interventions for other, nongenetic, chronic obstructive respiratory diseases.

## Methods

### Analysis of nasal epithelial cells.

The collection of nasal epithelial cells by brushing was previously described (26). The brush was immediately placed in 10% neutral buffered formalin and shipped to the laboratory, where cells were detached from the brush and deposited on silanized glass slides. After attachment (2–3 hours) in a humidified chamber, cells were treated for antigen retrieval with 10 mM citrate buffer and then permeabilized with 0.3% Triton X-100 in PBS for 5 minutes, blocked with 1% BSA in PBS (2 hours), and incubated overnight at 4°C with primary antibodies diluted in PBS containing 1% BSA (A7030, Sigma-Aldrich). Primary antibodies and dilutions were: rabbit anti-ATP12A (HPA039526, Sigma-Aldrich), 1:400; mouse IgG1 anti-MUC5AC (MA5-12178; Thermo Fisher Scientific), 1:200; and mouse IgG2B anti–acetylated tubulin (MilliporeSigma), 1:300. Samples were washed 3 times in PBS and incubated for 1 hour in the dark with the following secondary antibodies diluted 1:200 in PBS containing 1% BSA: goat anti–rabbit Alexa Fluor 488 (catalog A11008), goat anti–mouse IgG1 Alexa Fluor 546 (catalog A2112), and goat anti–mouse IgG2B Alexa Fluor 633 (catalog A21146) antibodies (all from Thermo Fisher Scientific). After 3 further washes in PBS, slides were mounted using Fluoroshield with DAPI (MilliporeSigma) to stain cell nuclei.

Immunofluorescence images were acquired with a laser scanning confocal microscope (TCS SPE; Leica Microsystems). For each sample, 200–500 cells were analyzed. To quantify ATP12A expression in the AM of cells, 2 regions of interest (ROIs) were selected: one on the AM and another placed halfway between the AM and the nucleus — i.e., the cytosol (C). ROI positioning was done in merged fluorescence and bright-field images to easily detect the AM in cells with low ATP12A expression. The mean fluorescence intensity of each ROI was calculated with the ImageJ software (NIH), and only cells with AM/C ratio higher than 2 were considered positive for ATP12A.

### Bronchial epithelial cell expansion and differentiation.

Human bronchial epithelial cells from CF patients (homozygous for F508del mutation) and non-CF individuals were provided by “Servizio Colture Primarie” of Italian Cystic Fibrosis Research Foundation (FFC). A detailed description of methods for the isolation and expansion of basal airway stem cells (p63^+^/ KRT5^+^) with a serum-free medium has been previously reported (11, 26). To further promote cell proliferation, the medium was supplemented with bone morphogenetic protein (BMP) antagonist (DMH-1, 1 μM), TGF-β antagonist (A 83-01, 1 μM), and the rho-associated protein kinase 1 (ROCK1) inhibitor (Y-27632, 10 μM) (58). After 5–6 passages, cells were seeded at high density on Snapwell (cc3801, Corning Costar) or Transwell (cc3470, Corning Costar) porous inserts. After 24 hours from seeding, the proliferative medium on the basolateral side was switched to differentiation medium PneumaCult ALI (Stemcell Technologies), whereas the medium on the apical side was removed to obtain the ALI condition. Epithelia were kept for at least 2–3 weeks to achieve full mucociliary differentiation. When needed, cells were treated basolaterally for 72 h with IL-17A (20 ng/mL, SRP3080, Sigma-Aldrich) plus/minus TNF-α (10 ng/mL, T6674, Sigma-Aldrich), IL-4 (10 ng/mL, 14269, Sigma-Aldrich), IFN-α (10 ng/mL, H6041, Sigma-Aldrich), IFN-γ (10 ng/mL, I3265, Sigma-Aldrich), IL-1β (10 ng/mL H6291, Sigma-Aldrich), or IL-6 (10 ng/mL, I1395, Sigma-Aldrich).

### Immunofluorescence of cultured epithelia.

Snapwell supports carrying differentiated bronchial epithelial cells were fixed in 10% neutral buffered formalin and then washed in PBS 3 times. After antigen retrieval with 10 mM citrate buffer, the samples were permeabilized with 0.3% Triton X-100 in PBS for 5 minutes, blocked with 1% BSA in PBS for 2 hours, and then incubated overnight at 4°C with primary antibodies diluted in PBS containing 1% BSA. The following antibodies and dilutions were used: rabbit anti-ATP12A (HPA039526, Sigma-Aldrich), 1:400; mouse IgG1 anti-MUC5AC (MA5-12178, Thermo Fisher Scientific), 1:200; mouse IgG2B anti–acetylated tubulin (T7451, Sigma-Aldrich), 1:300; anti-SCNN1A (HPA012743, Sigma-Aldrich), 1:200; and anti-SCNN1B (HPA015612, Sigma-Aldrich), 1:120. After 3 washes with PBS, cells were incubated with appropriate combinations of secondary fluorescent antibodies. After 3 further washes in PBS, slides were mounted using Fluoroshield with DAPI to stain cell nuclei. Images were acquired using a laser scanning confocal microscope (TCS SPE; Leica Microsystems).

### Western blot.

Differentiated epithelia on Snapwell inserts were lysed in RAS buffer containing Complete Protease Inhibitor Cocktail (Roche). Cell lysates were centrifuged at 14,500*g* at 4°C for 15 minutes. Supernatant protein concentration was calculated using the Bio-Rad DC Protein Assay. In total, 20 μg of lysates was resolved in gradient (4%–15%) Criterion TGX precast gels (Bio-Rad) and transferred to nitrocellulose membranes with a Trans-Blot Turbo system (Bio-Rad). After 2 hours of blocking in 5% milk in TBS-Tween 0.5%, membranes were incubated overnight with primary antibodies in 5 % milk in TBS-Tween 0.5%: anti-ATP12A (1:4000, HPA039526, Sigma-Aldrich) or anti-GAPDH (1:5000, MAB374, MilliporeSigma). After 3 washes in TBS-Tween 0.5%, secondary antibodies were incubated for 1 hour at room temperature in the dark: polyclonal anti–rabbit (catalog P0448) and anti–mouse (catalog P0447) HR-conjugated secondary antibodies (Dako, Agilent). After 3 further washes, the signals were subsequently visualized by chemiluminescence using the SuperSignal West Femto Substrate (Thermo Fisher Scientific). Signals were acquired with the Molecular Imager UVITEC Cambridge System. ATP12A and GAPDH band intensities were analyzed with ImageJ software (NIH).

### Short-circuit current recordings.

Snapwell supports carrying differentiated bronchial epithelia were mounted in Ussing-like vertical chambers (EM-CSYS-8, Physiologic Instruments). Both apical and basolateral chambers were filled with 5 mL of a Ringer bicarbonate solution containing (in mM): 126 NaCl, 0.38 KH_2_PO_4_, 2.13 K_2_HPO_4_, 1 CaCl_2_, 1 MgSO_4_, 24 NaHCO_3_, 10 glucose, and phenol red. Solution on both sides were bubbled with 5% CO_2_/95% air and kept at 37°C. The transepithelial voltage was clamped at 0 mV with an 8-channel voltage-clamp amplifier (VCC MC8, Physiologic Instruments) connected to apical and basolateral chambers via Ag/AgCl electrodes and agar bridges (1M KCl in 2% agar). The resulting short-circuited current from each channel was recorded with the Acquire & Analize 2.3 software (Physiologic Instruments).

### FRAP assay.

Epithelial cells were plated on the bottom part of “flipped” Snapwell inserts. After cell adhesion, Snapwell inserts were returned to their normal position and medium was added to the top part of the Snapwell (basolateral with respect to cells) and removed from the bottom part. Epithelia were allowed to differentiate (2–3 weeks). For experiments done in the presence of HCO_3_^–^, the basolateral medium was PneumaCult ALI. For experiments done under HCO_3_^–^-free conditions, the basolateral medium was Coon’s modification of F12 containing 20 mM HEPES (pH 7.4). The apical epithelial surface was stained with 5 μL PBS containing FITC-Dextran (70 kDa, 2 μg/mL, Thermo Fisher Scientific). After 3 hours, Snapwells with epithelia were mounted on the stage of a Nikon Eclipse Ti-E Spinning Disk inverted microscope within a chamber that allowed control of temperature (37°C) and atmosphere (humidified 5% CO_2_/95% air or pure air depending on the presence of bicarbonate in the basolateral medium). Images of the stained epithelial surface were sequentially acquired for 5 seconds before and 60 seconds after photobleaching of a preselected circular ROI (50 μm diameter). After normalization for the initial value, the recovery of fluorescence at 30 seconds from bleach pulse was measured.

### MicroParticle transport (MPT) assay.

Black micro beads (6 μm diameter; 24293-5 Polybead Black Dyed Microspheres, Polysciences) suspended in 2 μL PBS were added to the apical side of epithelia. Microbead movement was recorded with a Leica M205FA Stereo microscope. Microbeads velocity was calculated with ImageJ using Manual Tracking plug-in.

### Large-volume in situ pH assay.

This assay was used to measure net ATP12A activity in HCO_3_^–^-free conditions. In total, 60 μL of a modified PBS solution with low buffer capacity containing the pH-sensitive SNARF-1 probe coupled to 70 kDa dextran (0.1 mg/mL, D3304, Thermo Fisher Scientific) was added on the apical side of epithelia formed on Transwell inserts. The modified PBS solution had the following composition (in mM): 145 NaCl, 2.7 KCl, 0.81 Na_2_HPO_4_, 0.15 KH_2_PO_4_, 1 CaCl_2_, 0.5 MgCl_2_ (pH 7.35). The microplates carrying Transwell inserts were introduced in a FLUOstar Omega microplate reader (BMG LABTECH). Fluorescence was measured using single excitation (544 nm) and double emission (590 and 640 nm) at the beginning of treatment and every 60 minutes for 3 hours. The ratio of fluorescence emitted at 590 and 640 nm was converted to pH values using a calibration curve.

### Large-volume ex situ pH assay.

In total, 200 μL of PBS with/without compounds was added on the apical side of epithelia formed on Snapwell inserts. The saline solution was covered by 200 μL of mineral oil to avoid evaporation and CO_2_ exchange with atmosphere. The basolateral medium was PneumaCult ALI for experiments done in the presence of bicarbonate or Coon’s modification of F12 containing 20 mM HEPES (pH 7.4) for bicarbonate-free conditions. Epithelia were then incubated at 37°C in 5% CO_2_/95% air or pure air atmosphere. After 6 hours, the apical fluid was recovered, centrifuged (2,000 *g*, 1 minute, room temperature) to separate the 2 phases of mineral oil and PBS. Then, 50 μL of the aqueous phase from each sample plus 0.5 μL of SNARF-1 were rapidly transferred to the wells of 96-well microplates. The fluorescence was measured in a FLUOstar Omega microplate reader (BMG LABTECH) using single excitation (544 nm) and double emission (590 and 640 nm). The ratio of fluorescence emitted at 590 and 640 nm was converted to pH values using a calibration curve.

### Small-volume in situ pH assay.

In total, 5 μL of PBS containing the SNARF-1 dextran conjugate was spotted on the apical side of epithelia on Snapwell inserts. After staining, epithelia were incubated for 3 hours at 37°C. The basolateral medium was PneumaCult ALI medium (5% CO_2_/95% air atmosphere). After incubation, the fluorescence was measured with a FLUOstar Omega microplate reader (BMG LABTECH) using single excitation (544 nm) and double emission (590 and 640 nm). The ratio of fluorescence emitted at 590 and 640 nm was converted to pH values using a calibration curve.

### QuantSeq 3′ mRNA sequencing library preparation.

Analysis of epithelial transcriptome was done on 4 non-CF and 3 CF separate preparations in which epithelia were treated with/without IL-17/TNF-α. An additional set of data included 4 separate non-CF epithelia preparations treated with/without IL-4. Total RNA was extracted with Relia Prep RNA Cell Miniprep System (Z6011, Promega) according to the manufacturer’s instructions. RNA concentration was determined with the Qubit 2.0 fluorimetric Assay (Thermo Fisher Scientific) and adjusted at 10 ng/μL. Libraries were prepared from 125 ng of total RNA using the QuantSeq 3′ mRNA-Seq Library Prep Kit FWD for Illumina (Lexogen GmbH). Quality of libraries was assessed using screen tape High Sensitivity DNA D1000 (Agilent Technologies). The amplified fragmented cDNA of 300 bp in size was sequenced in single-end mode using the Illumina NovaSeq 6000 with a read length of 100 bp. Illumina NovaSeq base call (BCL) files were converted in fastq file through bcl2fastq (http://emea.support.illumina.com/content/dam/illuminasupport/documents/documentation/software_documentation/bcl2fastq/bcl2fastq2-v2-20-software-guide-15051736-03.pdf; version v2.20.0.422).

### QuantSeq 3′ mRNA sequencing data processing and analysis.

Sequence reads were trimmed using bbduk software (https://jgi.doe.gov/data-and-tools/software-tools/bbtools/bb-tools-user-guide/bbduk-guide/) to remove adapter sequences, poly-A tails, and low-quality end bases (regions with average quality below 6). Alignment was performed with STAR 2.6.0a (59) on hg38 reference assembly obtained from Cell Ranger website (https://support.10xgenomics.com/single-cell-gene-expression/software/release-notes/build#mm10_3.0.0; Ensembl assembly release 93). Expression levels of genes were determined with htseq-count (60) using Gencode/Ensembl gene model. Genes with an average number of CPM (counts per million) < 1 and percentage of duplicated reads > 20% were filtered out. Differential expression analysis was performed using edgeR (61), a statistical package based on generalized linear models, suitable for multifactorial experiments. The threshold for statistical significance chosen was FDR < 0.05. The Supplemental Table 1 includes the 151 genes significantly induced in CF and non-CF epithelia by IL-17/TNF-α. Gene Ontology (GO) and Functional Annotation Clustering analyses were performed on these 151 genes by using DAVID Bioinformatic Resources (62, 63) restricting the output to Biological Process terms (BP_FAT). The threshold for statistical significance of GOEA was FDR < 0.1 and enrichment score (i.e., the amount to which the genes in the cluster of Gene Ontology are overrepresented) was > 1.5.

### Accession codes.

The gene expression data were deposited in GEO with the SuperSeries accession no. GSE182958. The SuperSeries includes 3 data sets: the treatment with/without IL-4 on non-CF cells (GSE182955), the treatment with IL-17/TNF-α on CF cells (GSE182956), and the treatment with IL-17/TNF-α on non-CF (GSE182957).

### scRNA-Seq.

Bronchial epithelial cells were detached from Snapwell inserts as single cells through the following protocol: both apical and basolateral side were washed with PBS without Ca^2+^ and Mg^2+^, and they were then incubated for 30 minutes with Versene and for a further 15 minutes in 10× concentrated trypsin solution at 37°C. Cells were collected directly in New Zealand serum plus 10% DMSO, frozen for 24 hours at –80°C, and then stored in liquid nitrogen. For sequencing, single cells were suspended in phosphate buffered saline containing 0.04% BSA, filtered using a 40 μm cell strainer (Biologix), and counted with LUNA-II Automated Cell Counter (Logos Biosystems). The cell suspension was loaded onto the Chromium Single Cell G Chip Kit (10x Genomics) and run on the Chromium Single Cell Controller (10x Genomics) to generate single-cell gel bead emulsion, according to the manufacturer’s protocol. The single cell 3′ Library and Gel Bead Kit V3.1 (10x Genomics) was used to generate cDNA and the final libraries. The cDNA quality was assessed using high-sensitivity D5000 screen tape on Agilent 4200 TapeStation system (Agilent Technologies). Quality of libraries was assessed by using screen tape High sensitivity DNA D1000 (Agilent Technologies). Finally, the libraries were sequenced on Novaseq6000 sequencer (Illumina) according to manufacturer specifications. Sequencing was done by Next Generation Diagnostics.

Raw base call generated by sequencing was demultiplexed by using cellranger mkfastq (64) with standard parameters. Then, FASTQ files from cellranger mkfastq were aligned, filtered, and counted with cellranger count (64) with standard parameters. The output counted data were then used for downstream analysis using Seurat (4.0.4) (65). Only cells with mitochondrial rate < 15% were retained. Moreover, cells showing less than 500 genes and less than 1,000 unique molecular identifiers (UMIs) were discarded. Data were normalized using NormalizeData() function with LogNormalize method and scale factor = 10,000. Data were then scaled through the ScaleData() function, by taking all genes as features parameter. PCA dimensionality reduction was performed using RunPCA(), using, as features, the ones obtained by running the function VariableFeatures() on the data sets. Then, FindNeighbors(), FindClusters(), and the nonlinear dimensionality reduction RunUMAP() functions were used in order to obtain the 2D maps and the clusters for each analysis. Marker genes of each cluster were identified by using the FindAllMarkers() function using the Wilcoxon rank-sum test comparing cells in each cluster with all other cells in the data set.

### Data visualization.

Heatmaps and Venn diagrams were generated using custom annotation scripts. All graphs and figures were prepared with Igor Pro (WaveMetrics).

### Statistics.

Quantitative data are shown as scatter dot plots with mean ± SD. Each symbol in the plots represents the result of an independent experiment. To assess significant differences between groups of data, we first used the Kolmogorov-Smirnov test to evaluate normal distribution. For normally distributed data, we then used 2-tailed Student’s *t* test, in case of 2 groups, or 1-way ANOVA, for more than 2 groups. ANOVA was followed by Dunnett’s or Tukey’s post hoc tests, as appropriate. For data with nonnormal distribution, we used nonparametric tests: Mann-Whitney *U* for 2 groups and Kruskal-Wallis with Dunn’s test for more than 2 groups. Statistical analysis was done with PRISM software (GraphPad).

### Study approval.

The collection of nasal epithelial cells by brushing and the collection of human bronchi from explanted lungs to generate cultures of epithelial cells were approved by the relevant Ethical Committee with the registration nos. CER 28/2020 and ANTECER 042-09/07/2018.

## Author contributions

DG, MB, PS, MG, GC, and IS performed the experiments. FC and VT collected and processed samples. DG, MB, SS, and RDC analyzed data. DG, MB, PS, and LJVG prepared the figures. GP, GC, IS, and LJVG designed and supervised the study. DG and LJVG wrote the manuscript.

## Supplementary Material

Supplemental data

Supplemental table 1

## Version 1 10/11/2022

In-Press Preview

## Version 2 11/22/2022

Electronic publication

## References

[1] Knowles MR, Boucher RC (2002). Mucus clearance as a primary innate defense mechanism for mammalian airways. J Clin Invest.

[2] Bustamante-Marin XM, Ostrowski LE (2017). Cilia and mucociliary clearance. Cold Spring Harb Perspect Biol.

[3] Ridley C, Thornton DJ (2018). Mucins: the frontline defence of the lung. Biochem Soc Trans.

[4] Bartoszewski R (2017). Ion channels of the lung and their role in disease pathogenesis. Am J Physiol Lung Cell Mol Physiol.

[5] Bals R, Hiemstra PS (2004). Innate immunity in the lung: how epithelial cells fight against respiratory pathogens. Eur Respir J.

[6] Lambrecht BN (2019). The cytokines of asthma. Immunity.

[7] Andreakos E (2019). Lambda interferons come to light: dual function cytokines mediating antiviral immunity and damage control. Curr Opin Immunol.

[8] Higgins G (2015). Physiological impact of abnormal lipoxin A_4_ production on cystic fibrosis airway epithelium and therapeutic potential. Biomed Res Int.

[9] Kim CH (2009). Upregulation of MUC5AC gene expression by IL-4 through CREB in human airway epithelial cells. J Cell Biochem.

[10] Lachowicz-Scroggins ME (2010). Interleukin-13-induced mucous metaplasia increases susceptibility of human airway epithelium to rhinovirus infection. Am J Respir Cell Mol Biol.

[11] Scudieri P (2012). Association of TMEM16A chloride channel overexpression with airway goblet cell metaplasia. J Physiol.

[12] Gorrieri G (2016). Goblet cell hyperplasia requires high bicarbonate transport to support mucin release. Sci Rep.

[13] Hoegger MJ (2014). Impaired mucus detachment disrupts mucociliary transport in a piglet model of cystic fibrosis. Science.

[14] Garcia MA (2009). Normal mouse intestinal mucus release requires cystic fibrosis transmembrane regulator-dependent bicarbonate secretion. J Clin Invest.

[15] Gustafsson JK (2012). Bicarbonate and functional CFTR channel are required for proper mucin secretion and link cystic fibrosis with its mucus phenotype. J Exp Med.

[16] Pezzulo AA (2012). Reduced airway surface pH impairs bacterial killing in the porcine cystic fibrosis lung. Nature.

[17] Elborn JS (2016). Cystic fibrosis. Lancet.

[18] Kurkowiak M (2015). Recent advances in primary ciliary dyskinesia genetics. J Med Genet.

[19] Matsui H (1998). Evidence for periciliary liquid layer depletion, not abnormal ion composition, in the pathogenesis of cystic fibrosis airways disease. Cell.

[20] Button B (2012). A periciliary brush promotes the lung health by separating the mucus layer from airway epithelia. Science.

[21] Garnett JP (2011). Novel role for pendrin in orchestrating bicarbonate secretion in cystic fibrosis transmembrane conductance regulator (CFTR)-expressing airway serous cells. J Biol Chem.

[22] Kim D (2019). Pendrin mediates bicarbonate secretion and enhances cystic fibrosis transmembrane conductance regulator function in airway surface epithelia. Am J Respir Cell Mol Biol.

[23] Shei RJ (2018). The epithelial sodium channel (ENaC) as a therapeutic target for cystic fibrosis. Curr Opin Pharmacol.

[24] Scudieri P (2018). Increased expression of ATP12A proton pump in cystic fibrosis airways. JCI Insight.

[25] Shah VS (2016). Airway acidification initiates host defense abnormalities in cystic fibrosis mice. Science.

[26] Scudieri P (2020). Ionocytes and CFTR chloride channel expression in normal and cystic fibrosis nasal and bronchial epithelial cells. Cells.

[27] Vieira Braga FA (2019). A cellular census of human lungs identifies novel cell states in health and in asthma. Nat Med.

[28] Chen G (2019). IL-1β dominates the promucin secretory cytokine profile in cystic fibrosis. J Clin Invest.

[29] Rehman T (2020). TNFα and IL-17 alkalinize airway surface liquid through CFTR and pendrin. Am J Physiol Cell Physiol.

[30] Caputo A (2008). TMEM16A, a membrane protein associated with calcium-dependent chloride channel activity. Science.

[31] Danahay H (2002). Interleukin-13 induces a hypersecretory ion transport phenotype in human bronchial epithelial cells. Am J Physiol Lung Cell Mol Physiol.

[32] Galietta LJ (2002). IL-4 is a potent modulator of ion transport in the human bronchial epithelium in vitro. J Immunol.

[33] Gray T (2004). Regulation of MUC5AC mucin secretion and airway surface liquid metabolism by IL-1beta in human bronchial epithelia. Am J Physiol Lung Cell Mol Physiol.

[34] Rehman T (2021). Inflammatory cytokines TNF-α and IL-17 enhance the efficacy of cystic fibrosis transmembrane conductance regulator modulators. J Clin Invest.

[35] Gaillard EA (2010). Regulation of the epithelial Na+ channel and airway surface liquid volume by serine proteases. Pflugers Arch.

[36] Rossier BC, Stutts MJ (2009). Activation of the epithelial sodium channel (ENaC) by serine proteases. Annu Rev Physiol.

[37] Coote K (2009). Camostat attenuates airway epithelial sodium channel function in vivo through the inhibition of a channel-activating protease. J Pharmacol Exp Ther.

[38] Alvarez de la Rosa D (2002). Effects of aldosterone on biosynthesis, traffic, and functional expression of epithelial sodium channels in A6 cells. J Gen Physiol.

[39] Kabra R (2008). Nedd4-2 induces endocytosis and degradation of proteolytically cleaved epithelial Na+ channels. J Biol Chem.

[40] Debonneville C (2001). Phosphorylation of Nedd4-2 by Sgk1 regulates epithelial Na(+) channel cell surface expression. EMBO J.

[41] Lu M (2010). mTOR complex-2 activates ENaC by phosphorylating SGK1. J Am Soc Nephrol.

[42] Gleason CE (2015). mTORC2 regulates renal tubule sodium uptake by promoting ENaC activity. J Clin Invest.

[43] Haggie PM (2016). Inhibitors of pendrin anion exchange identified in a small molecule screen increase airway surface liquid volume in cystic fibrosis. FASEB J.

[44] Lee EH (2020). Inhibition of Pendrin by a small molecule reduces Lipopolysaccharide-induced acute lung injury. Theranostics.

[45] Li X (2021). V-Type ATPase mediates airway surface liquid acidification in pig small airway epithelial cells. Am J Respir Cell Mol Biol.

[46] Knight KK (2006). Liddle’s syndrome mutations increase Na+ transport through dual effects on epithelial Na+ channel surface expression and proteolytic cleavage. Proc Natl Acad Sci U S A.

[47] Rotin D, Staub O (2021). Function and regulation of the epithelial N^a^+ channel ENaC. Compr Physiol.

[48] Boucher RC (1988). Evidence for reduced Cl- and increased Na+ permeability in cystic fibrosis human primary cell cultures. J Physiol.

[49] Nagel G (2001). Non-specific activation of the epithelial sodium channel by the CFTR chloride channel. EMBO Rep.

[50] Collawn JF (2012). The CFTR and ENaC debate: how important is ENaC in CF lung disease?. Am J Physiol Lung Cell Mol Physiol.

[51] Itani OA (2011). Human cystic fibrosis airway epithelia have reduced Cl- conductance but not increased Na+ conductance. Proc Natl Acad Sci U S A.

[52] Mall M (1998). The amiloride-inhibitable N^a^+ conductance is reduced by the cystic fibrosis transmembrane conductance regulator in normal but not in cystic fibrosis airways. J Clin Invest.

[53] Adams KM (2014). IL-17A induces Pendrin expression and chloride-bicarbonate exchange in human bronchial epithelial cells. PLoS One.

[54] Lennox AT (2018). ATP12A promotes mucus dysfunction during Type 2 airway inflammation. Sci Rep.

[55] Hagner M (2021). IL-17A from innate and adaptive lymphocytes contributes to inflammation and damage in cystic fibrosis lung disease. Eur Respir J.

[56] Patel-Chamberlin M (2016). The role of epithelial sodium channel ENaC and the Apical Cl-/HCO3- exchanger pendrin in compensatory salt reabsorption in the setting of Na-Cl cotransporter (NCC) inactivation. PLoS One.

[57] Garland AL (2013). Molecular basis for pH-dependent mucosal dehydration in cystic fibrosis airways. Proc Natl Acad Sci U S A.

[58] Mou H (2016). Dual SMAD signaling inhibition enables long-term expansion of diverse epithelial basal cells. Cell Stem Cell.

[59] Dobin A (2013). STAR: ultrafast universal RNA-seq aligner. Bioinformatics.

[60] Anders S (2015). HTSeq--a Python framework to work with high-throughput sequencing data. Bioinformatics.

[61] Robinson MD (2010). edgeR: a Bioconductor package for differential expression analysis of digital gene expression data. Bioinformatics.

[62] Huang da W (2009). Bioinformatics enrichment tools: paths toward the comprehensive functional analysis of large gene lists. Nucleic Acids Res.

[63] Huang da W (2009). Systematic and integrative analysis of large gene lists using DAVID bioinformatics resources. Nat Protoc.

[64] Zheng GX (2017). Massively parallel digital transcriptional profiling of single cells. Nat Commun.

[65] Hao Y (2021). Integrated analysis of multimodal single-cell data. Cell.

