# Supplementary material for: Airway surface hyperviscosity and defective mucociliary transport by IL-17/TNF-α are corrected by β-adrenergic stimulus
Source: JCI Insight. 2022 Nov 22;7(22):e164944. doi: 10.1172/jci.insight.164944 (PMC9746827; doi:10.1172/jci.insight.164944)
Supplement: Supplemental data [file jciinsight-7-164944-s234.pdf]

Airway surface hyperviscosity and defective mucociliary transport by IL-17/TNF-α are corrected by beta-adrenergic stimulus

Daniela Guidone, Martina Buccirossi, Paolo Scudieri, Michele Genovese, Sergio Sarnataro, Rossella De Cegli, Federico Cresta, Vito Terlizzi, Gabrielle Planelles, Gilles Crambert, Isabelle Sermet, Luis J.V. Galietta

Supplementary Figures 1-7

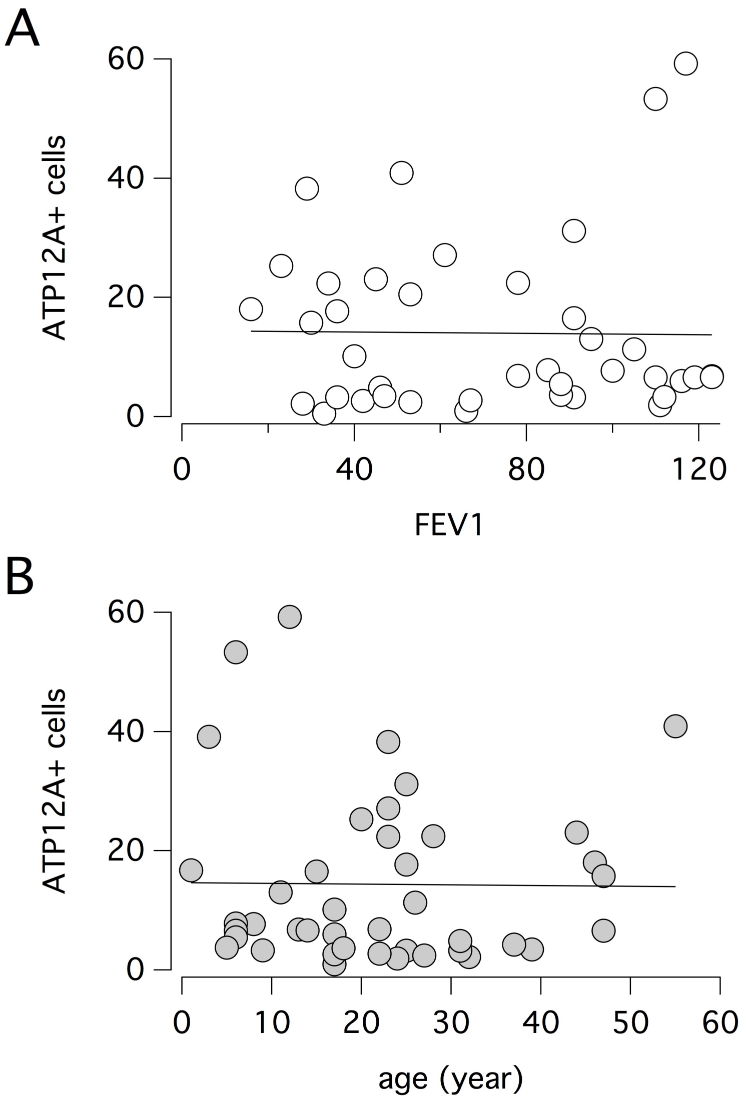

Supplementary Figure 1. Analysis of ATP12A expression in nasal epithelial cells freshly collected from CF patients. Each symbol reports the average value of a single patient. (**A**) Percentage of ATP12A+ cells vs. FEV1. (**B**) Percentage of ATP12A+ cells vs. age. Straight lines show the best linear fit of data.

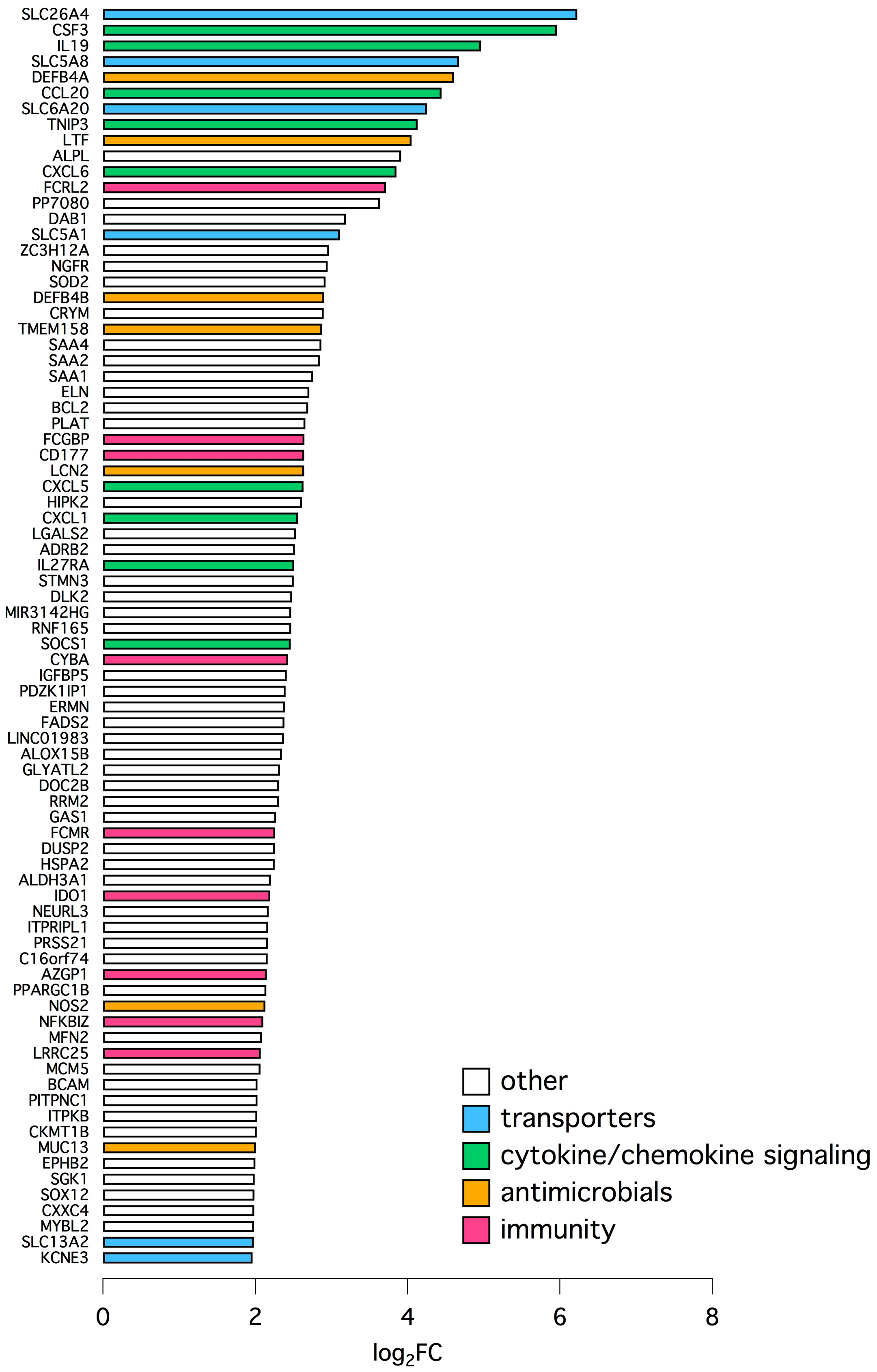

Supplementary Figure 2. Top 80 genes upregulated by IL-17/TNF-α combination in cultured bronchial epithelia. The bar graph shows the extent of expression of indicated genes as determined by bulk RNAseq in non-CF cultured bronchial epithelia. Color codes indicate gene function.

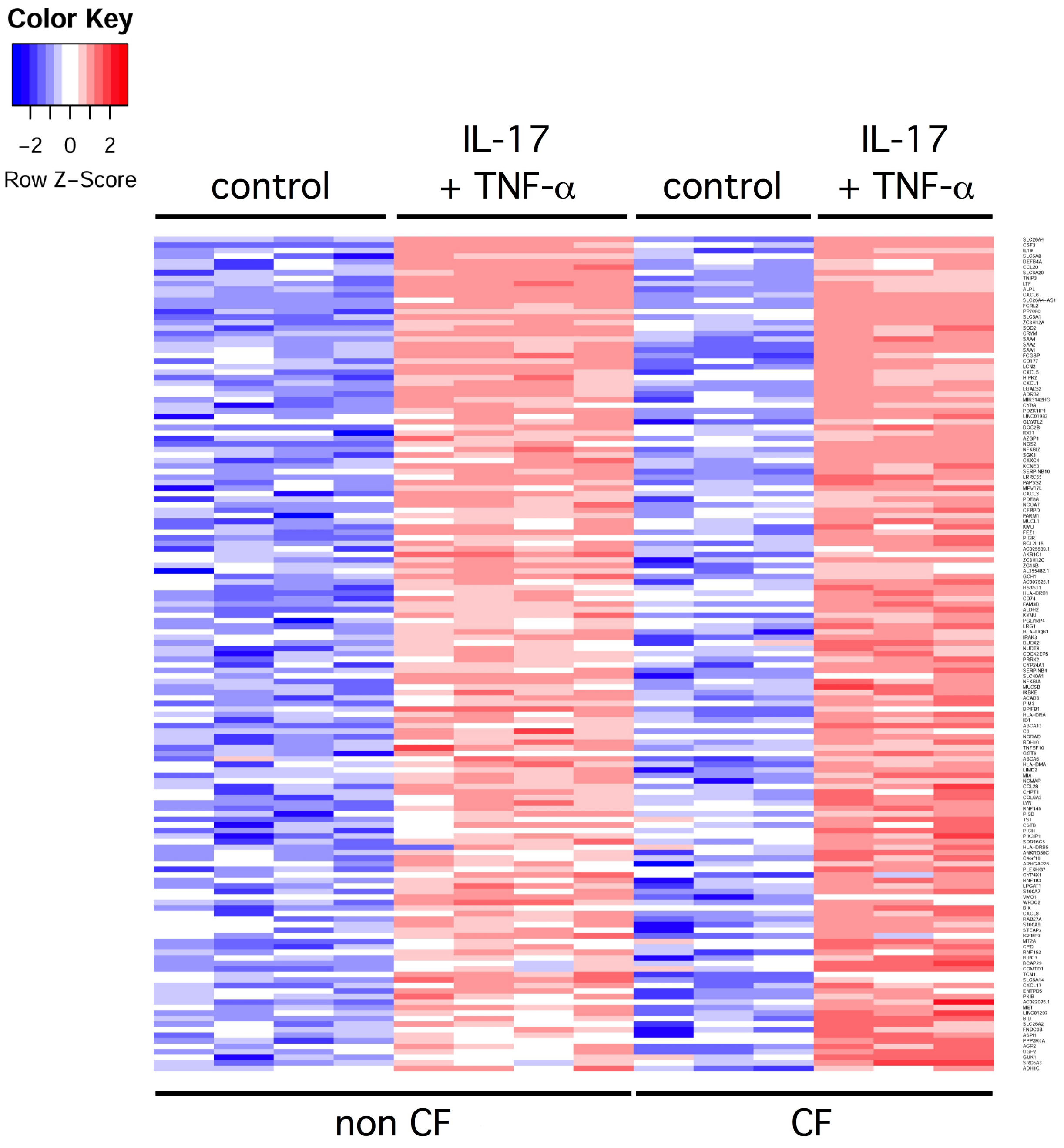

Supplementary Figure 3. Comparison of gene expression changes induced by IL-17/TNF-α in non-CF and CF cultured bronchial epithelia. The heat maps show the change in expression for the top 80 upregulated genes.

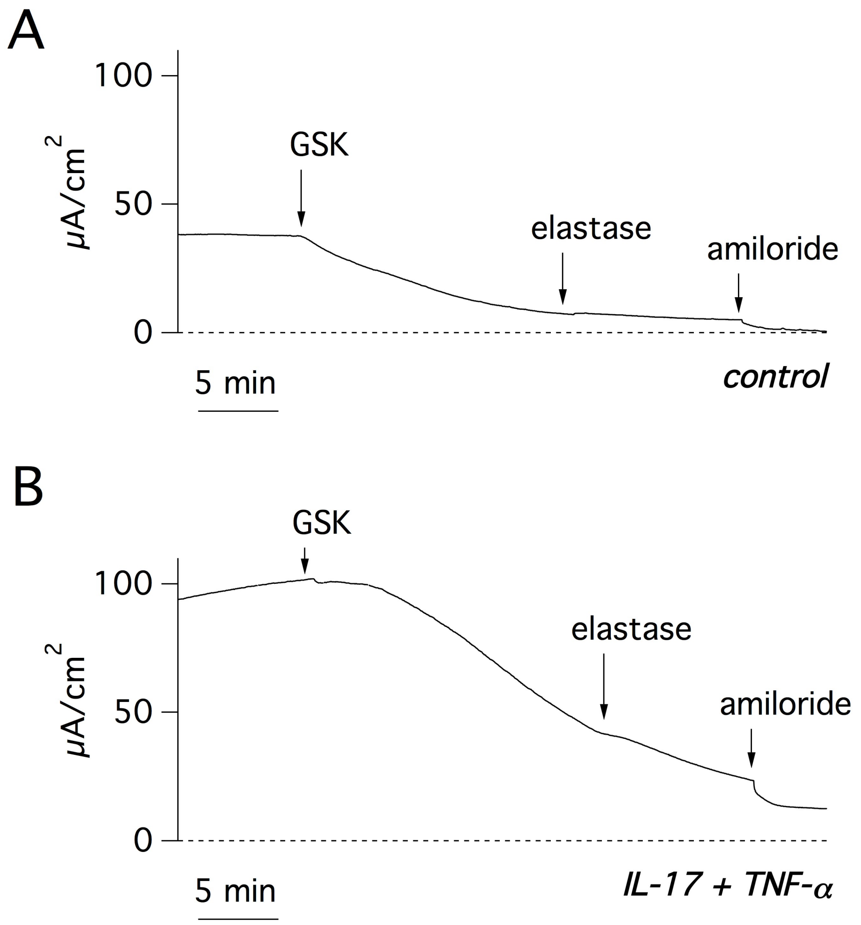

Supplementary Figure 4. Lack of elastase effect in epithelia treated with GSK650394. The figure shows representative short-circuit current recordings from cultured bronchial epithelia treated for 72 h in control conditions (**A**) or with IL-17/TNF-α (**B**).

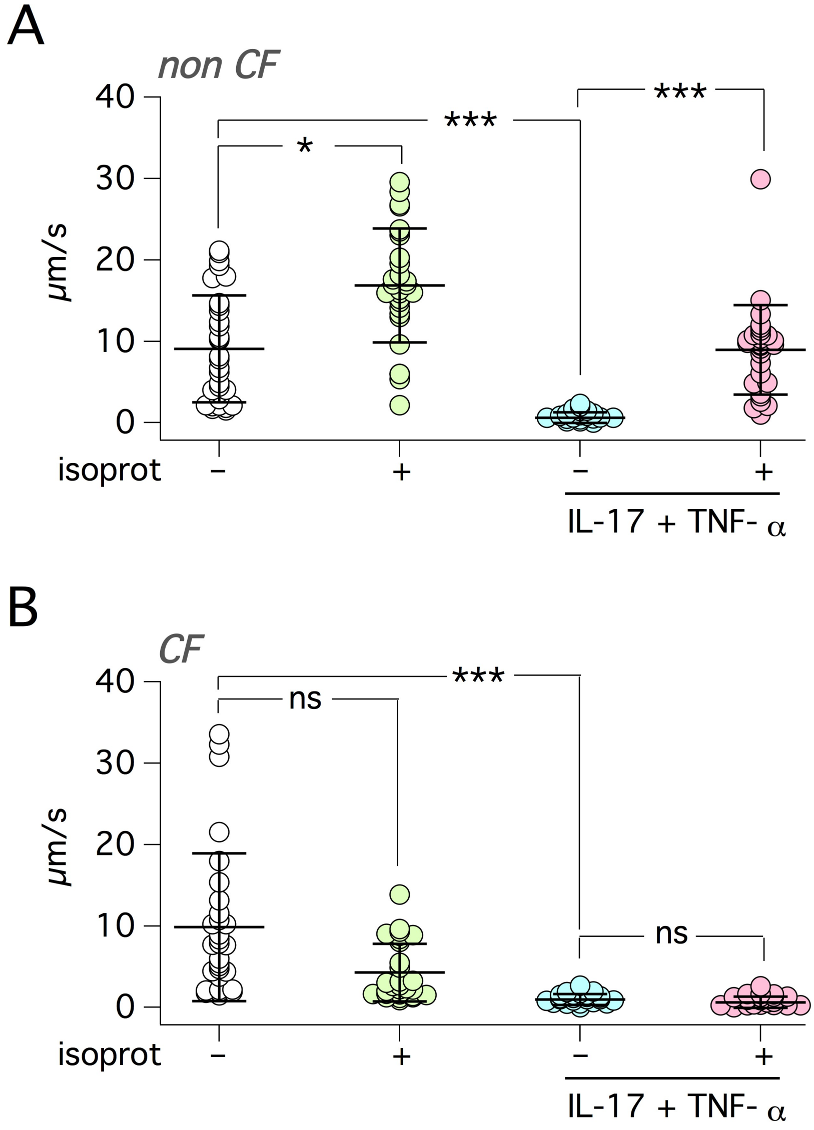

Supplementary Figure 5. Analysis of mucociliary transport by videomicroscopy. The graphs report the velocity of microbeads on non-CF (**A**) and CF (**B**) cultured bronchial epithelia. Epithelia were treated for 72 h with/without IL-17/TNF-α and then stimulated for 3 h with isoproterenol (100 nM). *, $p < 0.05$; **, $p < 0.01$; ***, $p < 0.001$; ns, not significant (Kruskal-Wallis and Dunn's test).

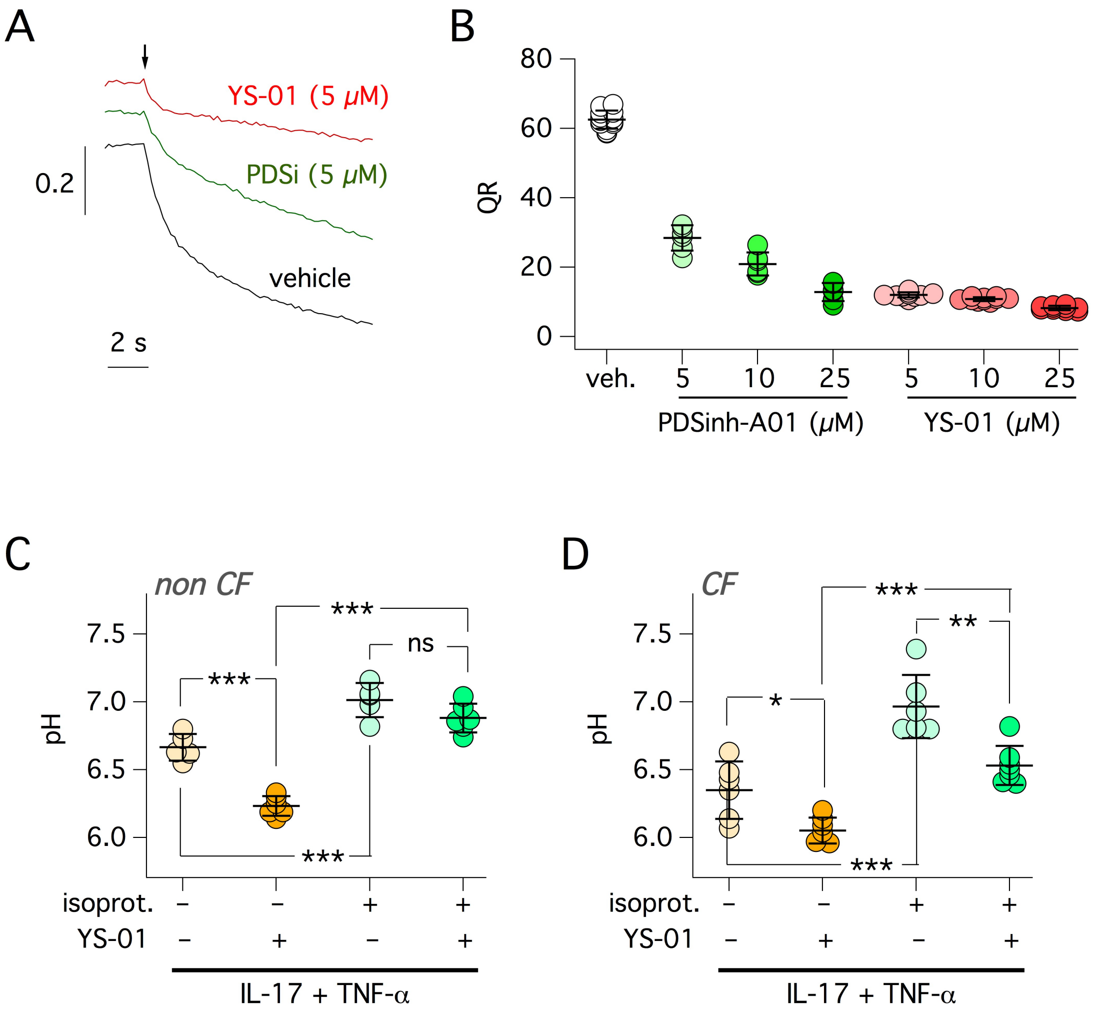

Supplementary Figure 6. Evaluation of SLC26A4 inhibitors. (**A**) Representative traces showing quenching of halide-sensitive yellow fluorescent protein (HS-YFP) caused by extracellular addition of I^- (arrow) in FRT cells expressing SLC26A4. SLC26A4 inhibitors (YS-01; PDS_{inh}-A01, PDSi) slow down I^- uptake and hence HS-YFP quenching. (**B**) Summary of data showing quenching rate (QR) of HS-YFP with vehicle and SLC26A4 inhibitors at the indicated concentrations. (**C**, **D**) Apical pH measured with the large volume ex situ assay in non-CF and CF cultured bronchial epithelia treated for 72 h with the IL-17/TNF-α combination. Where indicated, in the last 3 h, epithelia were stimulated with/without isoproterenol (100 nM) in the absence/presence of YS-01 (5 µM). *, $p < 0.05$; **, $p < 0.01$; ***, $p < 0.001$; ns, not significant (ANOVA with Tukey's post hoc test).

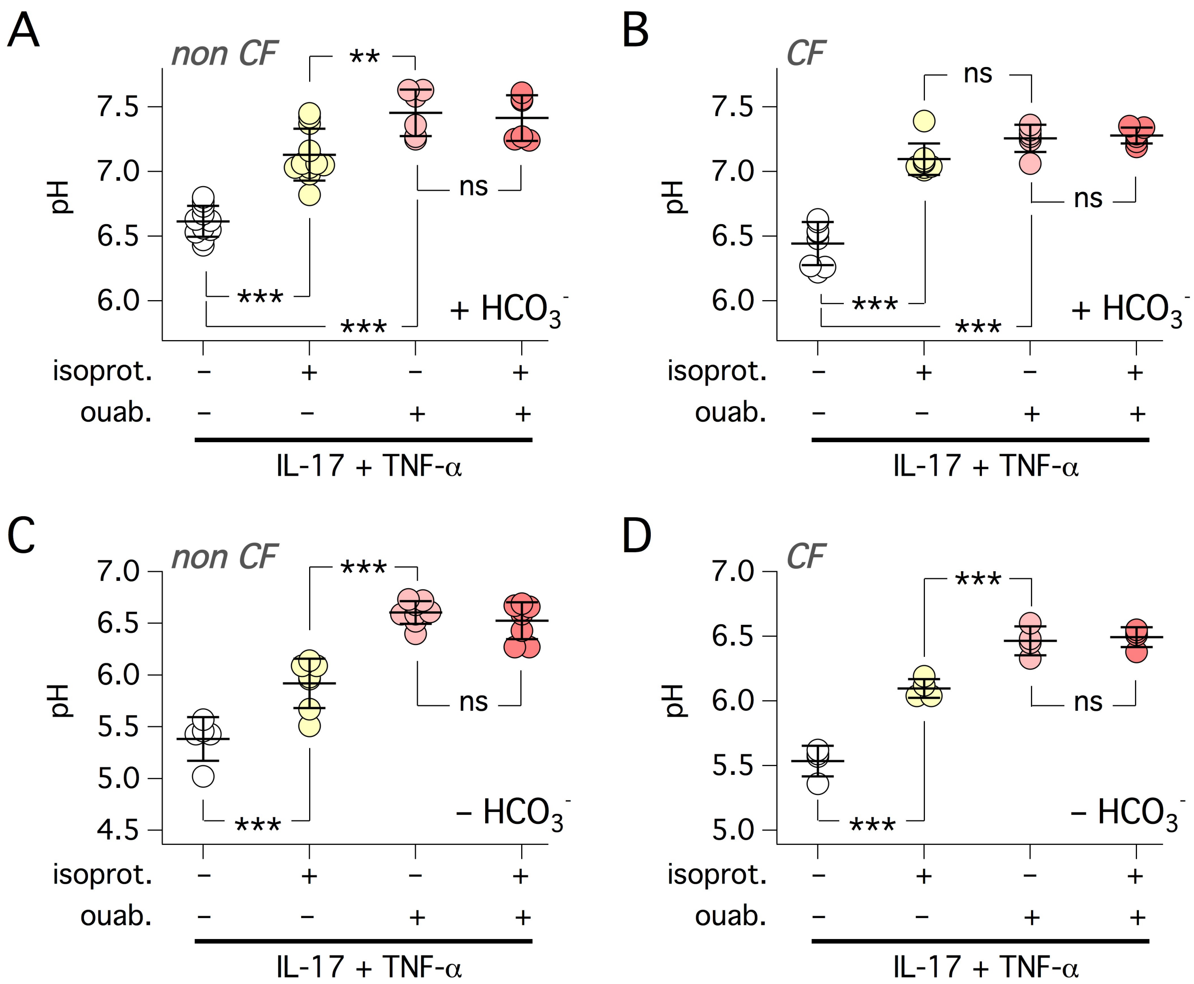

Supplementary Figure 7. Analysis of isoproterenol effect on apical pH. The graphs report the apical pH (large volume ex situ assay) measured in non-CF and CF bronchial epithelia kept in HCO_3^--rich (**A**, **B**) or HCO_3^--free (**C**, **D**) basolateral media. All epithelia were treated for 72 h with IL-17/TNF-α. Where indicated, epithelia were stimulated with basolateral isoproterenol (100 nM) in the absence/presence of ouabain (200 μM). **, $p < 0.01$; ***, $p < 0.001$; ns, not significant (ANOVA with Tukey's post hoc test).
